# A primordial synaptotagmin-independent function of complexin in regulated exocytosis

**DOI:** 10.1038/s41467-026-74947-4

**Published:** 2026-06-27

**Authors:** Xu Chen, Chun Wan, Jingyi Wu, Yan Ouyang, Harrison Puscher, Haijia Yu, Jingshi Shen

**Affiliations:** 1 Department of Molecular, Cellular and Developmental Biology, University of Colorado, Boulder, CO USA; 2 Jiangsu Key Laboratory for Molecular and Medical Biotechnology, College of Life Sciences, Nanjing Normal University, Nanjing, China; 3 Present Address: Department of Food Science and Nutrition, Hong Kong Polytechnic University, Hong Kong, China; 4 Present Address: Department of Biological Sciences, University of Southern California, Los Angeles, CA USA

**Keywords:** Membrane fusion, Exocytosis

## Abstract

In Ca²⁺-triggered exocytosis such as synaptic neurotransmitter release, vesicle fusion is tightly regulated by synaptotagmin (Syt) and complexin (Cpx), which together clamp partially assembled SNARE complexes to prevent premature fusion. However, Cpx is evolutionarily more ancient than Syt, suggesting that it may also regulate exocytosis independently of Syt. To test this possibility, we sought to identify an extant exocytic pathway that requires Cpx but naturally lacks Syt. Here, we uncovered such a pathway – hormone-triggered exocytosis of glucose transporters in adipocytes. In this pathway, Cpx acts exclusively as a positive regulator, accelerating the evoked phase of exocytosis without affecting basal fusion. Mechanistically, this Syt-independent activity depends on the central helix of Cpx for SNARE binding and on its C-terminal membrane-binding peptide, which remodels the lipid bilayer to promote exocytosis. Our findings support a model in which Cpx originally evolved to accelerate exocytosis independently of Ca²⁺, enabling rapid mobilization of exocytic cargoes in response to environmental cues. With the later emergence of Syt, Cpx acquired an additional role – acting in concert with Syt to regulate Ca²⁺-triggered exocytosis.

## Abstract (web summary)

This manuscript demonstrates a synaptotagmin-independent function of complexin in regulated exocytosis, which likely reflects complexin’s primordial role before the emergence of synaptotagmin.

## Introduction

Regulated exocytosis is a stimulus-dependent vesicle fusion event that rapidly relocates pre-synthesized molecules from intracellular storage vesicles to the cell surface or extracellular space^1–3^. This process underlies a wide range of physiological functions, including neuronal signaling, hormone secretion, nutrient homeostasis, and immune surveillance^1,4,5^. The vesicle fusion step in regulated exocytosis is catalyzed by a class of membrane-bound proteins known as soluble N-ethylmaleimide-sensitive factor attachment protein receptors (SNAREs)^5–8^. In SNARE-catalyzed fusion, vesicle-anchored v-SNAREs pair with target membrane-associated t-SNAREs to assemble into a four-helix trans-SNARE complex that pulls the two membranes into close apposition and drives fusion^9–13^.

A well-characterized regulated exocytic pathway is Ca²⁺-triggered synaptic exocytosis—the fusion of synaptic vesicles with the plasma membrane at the chemical synapse^6,14–16^. Upon the arrival of an action potential at the presynaptic terminal, voltage-gated calcium channels open, allowing a rapid influx of Ca²⁺ ions^17^. This Ca²⁺ surge triggers the fusion of synaptic vesicles with the plasma membrane, leading to the release of neurotransmitters into the synaptic cleft^16,18^. In this pathway, the t-SNAREs are syntaxin-1 and SNAP-25, whereas VAMP2/synaptobrevin-2 serves as the v-SNARE^6,19^. The Ca²⁺ sensor in synaptic exocytosis is the synaptotagmin (Syt) family, membrane-anchored proteins with tandem Ca²⁺-binding domains that regulate vesicle fusion by interacting with both SNAREs and membrane bilayers^3,20,21^.

Besides Syt, Ca²⁺-triggered synaptic exocytosis also involves complexin (Cpx), a family of small SNARE-binding proteins^22–28^. Although Cpx does not directly bind Ca²⁺, it is essential for the tight Ca²⁺ regulation of synaptic vesicle fusion^19,23,29^. Extensive studies across diverse in vivo and in vitro systems have demonstrated that a critical role of Cpx is to cooperate with Syt to clamp a partially zippered *trans*-SNARE complex, thereby stabilizing a primed state that arrests vesicle fusion^19,30–35^. Upon Ca²⁺ influx, Ca²⁺ binding to Syt induces a conformational change that relieves this arrest, allowing SNAREs to fully zipper and drive vesicle fusion. Cpx has also been proposed to positively regulate synaptic vesicle fusion^36–40^, although it remains unclear whether this activity is mechanistically linked to its Syt-dependent role or represents a distinct function^41^. Beyond synaptic exocytosis, Syt and Cpx also regulate other Ca²⁺-triggered exocytic pathways, including insulin secretion from pancreatic β cells, mast cell degranulation, and sperm acrosomal exocytosis, which are thought to operate through mechanisms similar to those in neurons^2,42–47^.

Intriguingly, although Cpx functions in concert with Syt during Ca²⁺-triggered exocytosis, Cpx is evolutionarily more ancient than Syt^48,49^. It emerged in unicellular organisms before the rise of metazoans, whereas Syt appeared later along the lineage of multicellular animals^48,49^. For instance, the choanoflagellate *Monosiga brevicollis*, a representative unicellular holozoan, expresses Cpx but lacks Syt^48–51^. This separation in evolutionary timing suggests that Cpx may also play a role in exocytosis independently of Syt. To determine whether such a Syt-independent function exists, we sought to identify an extant exocytic pathway that requires Cpx but naturally lacks Syt. Here, we uncovered such a pathway in adipocytes: insulin-triggered exocytosis of the glucose transporter type 4 (GLUT4), which depends on a single Cpx isoform, Cpx2, but not on any Syt isoforms. In this Syt-independent exocytic pathway, Cpx2 functions solely as a fusion accelerator. Its stimulatory activity is confined to the evoked phase of exocytosis and does not arrest basal vesicle fusion. At the molecular level, this Syt-independent function of Cpx2 requires its interaction with GLUT4 exocytic SNAREs and remodeling of the membrane bilayer through two distinct domains. These findings support a model that Cpx initially evolved to accelerate exocytosis independently of Ca²⁺, enabling rapid mobilization of exocytic cargoes in response to environmental cues. With the emergence of Syt, Cpx acquired an additional role – acting in concert with Syt to control Ca²⁺-triggered exocytosis – while its primordial Syt-independent function has been retained.

## Results

### Identification of adipocytes as a candidate system for investigating Syt-independent Cpx function

**Fig. 1:**
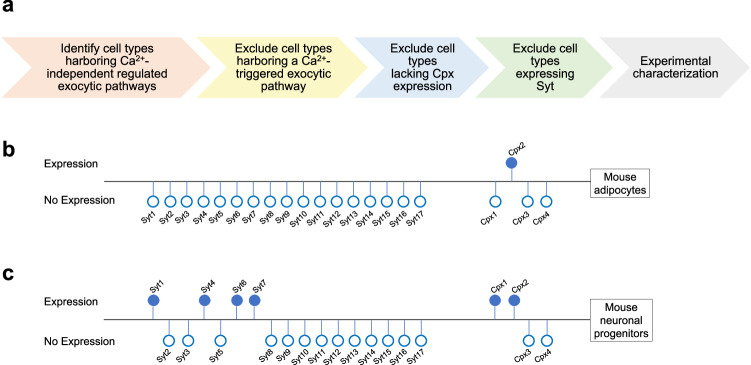
Mouse adipocytes express a single Cpx but no Syt. **a** Workflow for identifying regulated exocytic pathways that require Cpx but not Syt, screening all mouse and human cell types reported to harbor a regulated exocytic pathway^1,112,113^. **b** Expression patterns of Syt and Cpx isoforms in mouse adipocytes based on proteomic analysis, as shown in Supplementary Data 1. In this work, mouse adipocytes were differentiated from a preadipocyte cell line. **c** Expression patterns of Syt and Cpx isoforms in Cath.a-differentiated (CAD) cells based on proteomic analysis, as shown in Supplementary Data 2. CAD cells are a mouse catecholaminergic neuronal progenitor cell line that can be differentiated into neurons^65,114^.

To determine the Syt-independent function of Cpx, we first sought to identify a naturally occurring exocytic pathway that requires Cpx but lacks Syt. While choanoflagellates lack Syt, their exocytic pathways remain experimentally intractable. Alternatively, Syt-deficient neurons can be generated by deleting Syt-encoding genes, but such engineered systems do not capture native Syt-independent Cpx activity in a physiologically relevant context. We thus systematically searched for cell types that execute Cpx-dependent regulated exocytosis without involving any Syt isoforms (Fig. 1a). To identify such a system, we applied four stringent criteria: (1) the cell type harbors a regulated exocytic pathway triggered by a signal other than Ca²⁺; (2) no Syt-dependent Ca²⁺-triggered exocytic pathway is known in this cell type; (3) the cell type expresses Cpx; and (4) the cell type does not express any Syt isoform (Fig. 1a).

Among the cell types we examined, mammalian adipocytes emerged as a strong candidate. These cells possess a well-established regulated exocytic pathway—insulin-triggered GLUT4 exocytosis—in which vesicle fusion is controlled by insulin-dependent phosphorylation events rather than by Ca²⁺ ^1,52,53^. Other exocytic pathways in adipocytes are less well characterized, but none is known to be directly triggered by Ca²⁺ in a Syt-dependent manner, as occurs in synaptic exocytosis. A mammalian genome encodes four Cpx isoforms (Cpx1-4) and 17 Syt isoforms (Syt1-17)^54^. Cpx1 and Cpx2 are broadly expressed, whereas Cpx3 and Cpx4 are restricted to specific neuronal and retinal populations^3,23,25,37,55,56^. Our mass spectrometry (MS)-based proteomic analysis revealed that mouse adipocytes express Cpx2 but no other Cpx isoforms (Fig. 1b and Supplementary Data 1). Strikingly, none of the 17 Syt isoforms was detected in mouse adipocytes (Fig. 1b and Supplementary Data 1), consistent with earlier proteomic datasets^55,57^. In contrast, neuronal progenitor cells analyzed in parallel expressed multiple Syt isoforms (Fig. 1c and Supplementary Data 2), demonstrating that the absence of Syts in adipocytes was not due to technical limitations of MS. Immunoblotting confirmed the absence of exocytic Syts in adipocytes and their presence in neuronal progenitors (Supplementary Fig. 1).

The absence of Syt in mouse adipocytes agrees with a lack of known Syt-dependent Ca²⁺-triggered exocytic pathways in this cell type^1^. Although we cannot fully exclude the possibility that a Syt isoform is expressed below the threshold of proteomic detection, such trace expression is unlikely to be biologically significant. The functions of vesicle fusion regulators like Syts require stoichiometric interactions with SNAREs, making sub-detectable levels functionally irrelevant^6,20,58^. Together, these analyses establish mouse adipocytes as a promising physiological system in which to investigate the Syt-independent function of Cpx.

### Cpx2 plays a stimulatory role in insulin-triggered GLUT4 exocytosis in adipocytes

In the GLUT4 exocytic pathway, the glucose transporter GLUT4 is sequestered in intracellular storage vesicles under the basal condition^59–63^. Insulin stimulation induces phosphorylation of exocytic regulators, promoting the fusion of GLUT4-containing vesicles with the plasma membrane^64–67^. Once on the surface, GLUT4 promotes glucose uptake for energy production or storage, a process essential for blood glucose homeostasis and impaired in insulin resistance and type 2 diabetes^1,53,68–72^. Cpx2 has been implicated in GLUT4 exocytosis in skeletal muscle^24^, but muscle cells express Syts and harbor Ca²⁺-triggered exocytic pathways^24,73–75^, leaving open the possibility of Syt involvement. In contrast, adipocytes naturally lack Syts (Fig. 1), providing an ideal system to investigate the Syt-independent function of Cpx and to dissect its underlying molecular mechanism in a physiologically relevant context.

**Fig. 2:**
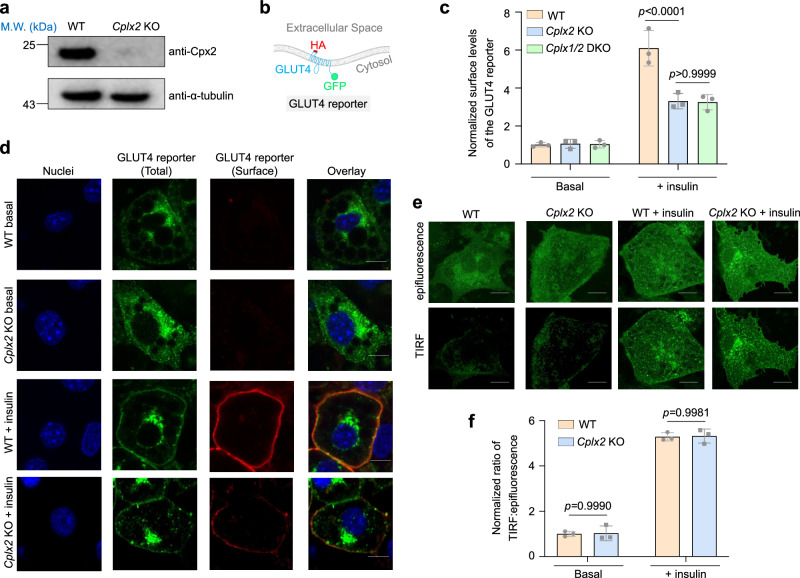
Cpx2 plays a positive role in insulin-triggered GLUT4 exocytosis in adipocytes. **a** Representative immunoblots from three independent experiments showing the expression of the indicated proteins in WT and *Cplx2* KO mouse adipocytes. M.W. molecular weight. **b** Diagram of the GLUT4 reporter^105,115^. **c** Normalized surface levels of the GLUT4 reporter in WT, *Cplx2* KO, and *Cplx1/2* DKO adipocytes. The cells were either untreated or treated with 100 nM insulin for 30 min before surface reporters were stained using anti-HA antibodies and APC-conjugated secondary antibodies. To calculate normalized surface levels of the reporter, mean APC fluorescence was divided by mean GFP fluorescence. Gating strategies and statistical measures are provided in Supplementary Figs. 3 and 4, respectively. In all figures, data normalization was performed by setting the mean value of WT data points without insulin stimulation as 1, and all data points, including WT ones, were normalized to that mean value. Data are presented as mean ± SD of three biological replicates. *p* values were calculated using one-way ANOVA and Tukey’s multiple comparison test. **d** Representative confocal images from three independent experiments showing the localization of the GLUT4 reporter in unpermeabilized WT and *Cplx2* KO adipocytes. The cells were either untreated or treated with 100 nM insulin for 30 min before surface GLUT4 reporters were labeled using anti-HA antibodies and Alexa Fluor 568-conjugated secondary antibodies, and visualized under a confocal microscope. Scale bars, 10 μm. **e** Representative epifluorescence and TIRF microscopy images of the GLUT4 reporter from three independent experiments in WT and *Cplx2* KO adipocytes. Cells were treated with 20 nM insulin for 20 min before imaging. Scale bars, 10 μm. **f** Normalized TIRF/epifluorescence ratio of GLUT4 reporters in WT and *Cplx2* KO adipocytes. TIRF signals represent plasma membrane–proximal GLUT4 reporters, whereas epifluorescence signals represent total GLUT4 reporters. Cells were cultured and imaged as in (**e**). Data were normalized as in (**c**). Data are presented as mean ± SD of three biological replicates. *p* values were calculated using two-way ANOVA with Tukey’s multiple comparison test.

To assess the role of Cpx2, we deleted the Cpx2-encoding *Cplx2* gene in mouse adipocytes using CRISPR genome editing (Fig. 2a). Surface and total levels of GLUT4 reporters were measured using flow cytometry (Fig. 2b, c). We observed that insulin-triggered GLUT4 exocytosis was markedly reduced in *Cplx2* knockout (KO) cells, whereas basal surface GLUT4 levels prior to insulin stimulation remained unchanged (Fig. 2b, c). The magnitude of this reduction was comparable to the deletion of known key regulators of GLUT4 exocytosis, such as Rab10 and the exocyst complex^66,76,77^. Cpx1, another broadly expressed isoform, was not detected in adipocytes (Fig. 1b and Supplementary Data 1). To exclude the possibility of residual Cpx1 activity, we generated a double knockout (DKO) of *Cplx1* and *Cplx2*, which showed no further reduction of insulin-triggered GLUT4 exocytosis compared to *Cplx2* KO alone (Fig. 2c and Supplementary Figs. 2–4). These data indicate that insulin-triggered GLUT4 exocytosis requires Cpx2, but not Cpx1, and further support the conclusion that proteins expressed below proteomic detection levels do not contribute appreciably to a vesicle fusion pathway.

We next visualized GLUT4 exocytosis directly using confocal microscopy. Consistent with flow cytometry results, insulin-triggered GLUT4 exocytosis was strongly reduced in *Cplx2* KO cells, whereas basal surface levels of GLUT4 remained unchanged (Fig. 2d). Total levels of the GLUT4 reporter were unchanged (Supplementary Fig. 5), indicating that Cpx2 regulates GLUT4 localization rather than overall expression. Notably, confocal and total internal reflection fluorescence (TIRF) microscopy showed that GLUT4 migration to the vicinity of the plasma membrane occurred normally in *Cplx2* KO cells (Fig. 2d–f), indicating that Cpx2 is not required for vesicle trafficking to the plasma membrane but instead promotes a late stage of the exocytic pathway.

Insulin signaling and adipocyte differentiation remained intact in *Cplx2* KO cells (Supplementary Fig. 6). Because these processes depend on constitutive exocytosis for the surface delivery of other membrane proteins such as the insulin receptor^52,78^, these results suggest that Cpx2 does not function as a general regulator of exocytosis. While adipocytes lack Syts (Fig. 1b), our MS analysis detected Sytl2 (also known as Slp2), a Syt-like protein (Supplementary Data 1). However, unlike Syts, Sytl2 does not bind Ca²⁺ or regulate fusion^79^, and deletion of *Sytl2* did not affect insulin-triggered GLUT4 exocytosis (Supplementary Fig. 7). Thus, Sytl2 is not involved in the GLUT4 exocytic pathway.

Together, these genetic experiments demonstrate that Cpx2 stimulates the evoked phase of the GLUT4 exocytic pathway in adipocytes. The asymmetric effects of Cpx2 on basal GLUT4 surface levels suggest that Cpx2 is not a constitutive driver of basal exocytosis. Instead, our data support a model in which Cpx2 acts at a late stage of the exocytic pathway that is engaged when vesicles become fusion-competent, consistent with a conditionally activated role downstream of insulin-dependent priming steps.

### Cpx does not arrest GLUT4 vesicle fusion prior to insulin induction

**Fig. 3:**
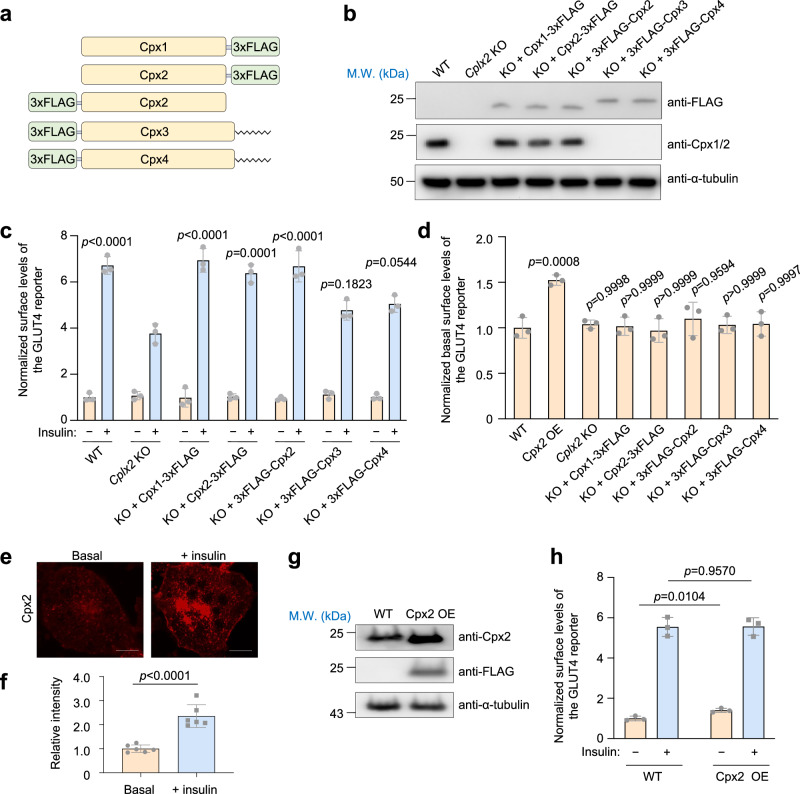
Cpx2 does not arrest GLUT4 vesicle fusion prior to insulin stimulation. **a** Diagrams of 3xFLAG-tagged human Cpxs. **b** Representative immunoblots from three independent experiments showing the expression of the indicated proteins. **c** Normalized surface levels of the GLUT4 reporter in the indicated mouse adipocytes. Cells were either untreated or treated with 100 nM insulin for 30 min before surface levels of the GLUT4 reporter were measured using flow cytometry. Data are presented as mean ± SD of three biological replicates. *p* values were calculated using one-way ANOVA and Tukey’s multiple comparison test. Data of insulin-treated samples were compared to those of insulin-treated *Cpx2* KO cells. **d** Normalized basal surface levels of the GLUT4 reporter in the indicated mouse adipocytes without insulin stimulation. Data are presented as mean ± SD of three biological replicates. *p* values were calculated using one-way ANOVA and Tukey’s multiple comparison test. **e** Representative TIRF microscopy images of Cpx2 in WT adipocytes from three independent experiments. The cells were either untreated or treated with 20 nM insulin for 20 min before the cells were fixed and permeabilized. Cells were immunostained for Cpx2 using anti-Cpx2 antibodies and Alexa Fluor 568-conjugated secondary antibodies, and imaged by TIRF microscopy. Scale bars, 10 μm. **f** Normalized levels of Cpx2 in TIRF fields. Adipocytes were cultured and imaged as in (**e**). Six fields were analyzed for each sample. Error bars indicate SD. *p* values were calculated using two-sided Student’s *t* test. **g** Representative immunoblots from three independent experiments showing the expression of the indicated proteins. **h** Normalized surface levels of the GLUT4 reporter in WT adipocytes with or without Cpx2 overexpression. The cells were either untreated or treated with 100 nM insulin for 30 min before surface levels of GLUT4 reporters were measured using flow cytometry. Data are presented as mean ± SD of three biological replicates. *p* values were calculated using two-sided Student’s *t* test.

Next, we examined whether other Cpx isoforms can support GLUT4 exocytosis, even though they are not normally expressed in mouse adipocytes. We reasoned that such a comparative analysis would provide insights into functional similarities and differences among Cpx isoforms. As expected, expression of a Cpx2 rescue construct at endogenous levels fully restored insulin-triggered GLUT4 exocytosis in *Cplx2* KO adipocytes (Fig. 3a–c). Similarly, Cpx1 expression fully rescued insulin-triggered GLUT4 exocytosis (Fig. 3a–c), indicating that Cpx1 and Cpx2 are functionally equivalent in regulating this pathway. By contrast, neither Cpx3 nor Cpx4 was able to restore insulin-triggered GLUT4 exocytosis when expressed at levels comparable to Cpx2 (Fig. 3a–c). Thus, Cpx3 and Cpx4 have functionally diverged from Cpx1 and Cpx2 in regulating exocytosis.

In Ca²⁺-triggered exocytosis, Cpx arrests basal vesicle fusion by acting in concert with Syt to clamp partially zippered SNARE complexes^3,19,31,80^. If Cpx also played such a clamping role in the GLUT4 exocytic pathway, its deletion would be expected to elevate surface levels of GLUT4 prior to insulin stimulation. However, basal surface GLUT4 levels remained unchanged in Cplx2 KO adipocytes, as well as in cells rescued with any of the Cpx isoforms (Figs. 2c, d and 3c, d), indicating that Cpx does not arrest vesicle fusion in the basal state. Using TIRF microscopy, we found that Cpx2 translocated to the vicinity of the plasma membrane upon insulin induction (Fig. 3e, f and Supplementary Fig. 8), confirming a stimulatory role of Cpx2 during the insulin-triggered phase of GLUT4 exocytosis rather than clamping fusion at the resting state. To further test this conclusion, we overexpressed Cpx2 in WT adipocytes to about three-fold of its endogenous level (Fig. 3g). Overexpression of Cpx2 increased basal surface GLUT4 instead of suppressing it (Fig. 3d, h), providing additional evidence that Cpx2 does not exert an inhibitory function in the GLUT4 exocytic pathway.

Together, these data demonstrate that Cpx2 does not act as a fusion clamp in the GLUT4 exocytic pathway but instead functions solely to accelerate exocytosis, enabling efficient GLUT4 translocation upon insulin stimulation.

### The Syt-independent function of Cpx2 requires its central helix and C-terminal domain

**Fig. 4:**
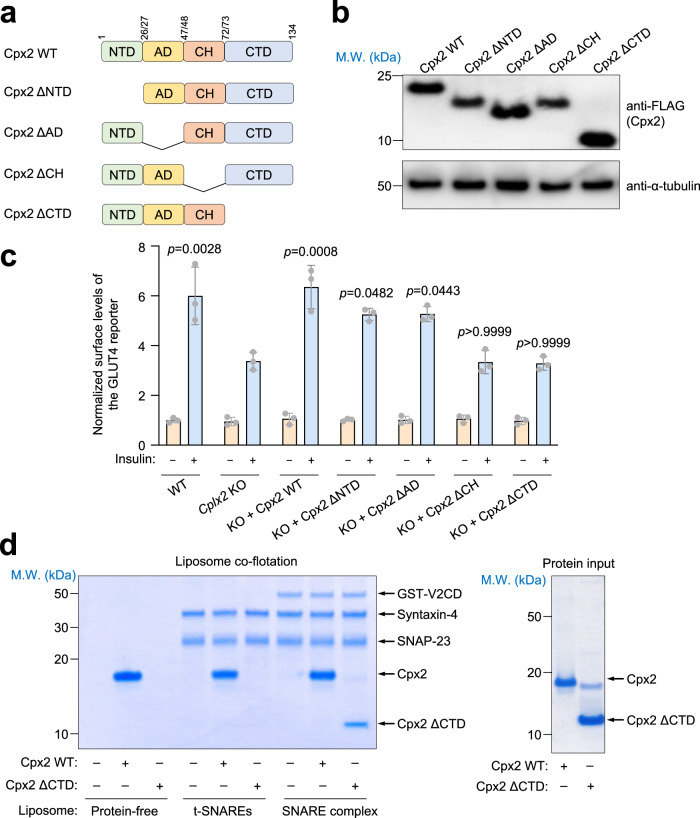
The Syt-independent function of Cpx2 in regulated exocytosis requires its CH and CTD. **a** Diagrams of WT and mutant human Cpx2 proteins. **b** Representative immunoblots from three independent experiments showing the expression of the indicated proteins. **c** Normalized surface levels of the GLUT4 reporter in the indicated mouse adipocytes. The cells were either untreated or treated with 100 nM insulin for 30 min before surface levels of the GLUT4 reporter were measured using flow cytometry. Data are presented as mean ± SD of three biological replicates. Data of insulin-treated samples were compared to those of insulin-treated *Cplx2* KO cells. *p* values were calculated using one-way ANOVA and Tukey’s multiple comparison test. **d** Representative liposome co-flotation assays from three independent experiments showing the binding of Cpx2 to the indicated liposomes. The t-SNARE liposomes contained syntaxin-4 and SNAP-23, whereas the SNARE complex liposomes were prepared by incubating t-SNARE liposomes with 5 μM GST-V2CD (the cytosolic domain of VAMP2). Liposomes were incubated with 10 μM WT or mutant Cpx2 at 37 °C for 1 h, then subjected to Nycodenz density gradient centrifugation to separate bound from unbound proteins. Liposome fractions were collected and analyzed using SDS-PAGE and Coomassie blue staining (left). Input Cpx proteins are shown at the right.

The GLUT4 exocytic pathway offers a unique physiological context in which to define the molecular mechanism of Syt-independent Cpx function. We began by mapping the domains of Cpx2 required for regulating the GLUT4 exocytic pathway. Cpx2 possesses four domains—an N-terminal domain (NTD), an accessory domain (AD), a central helix (CH), and a C-terminal domain (CTD) (Fig. 4a)^25,36,81^. Cpx2 mutants lacking each of these domains were individually expressed in *Cplx2* KO adipocytes and their ability to support insulin-triggered GLUT4 exocytosis was measured. We observed that deletion of CH or CTD abolished the stimulatory function of Cpx2 in the GLUT4 exocytic pathway (Fig. 4b, c). By contrast, deletion of NTD or AD reduced but did not abolish insulin-triggered GLUT4 exocytosis (Fig. 4b, c), suggesting that these domains play a modulatory rather than essential role. None of the Cpx2 mutants altered basal surface GLUT4 levels when expressed in *Cplx2* KO cells, indicating that they were not converted into inhibitors of GLUT4 exocytosis and reinforcing that Cpx2 functions solely as an accelerator in the exocytic pathway. Together, these data demonstrate that both the CH and CTD are essential to the Syt-independent function of Cpx2 in insulin-triggered GLUT4 exocytosis.

The function of a vesicle fusion regulator usually involves interactions with SNAREs, the membrane bilayer, or both^5,16^. Previous biochemical studies showed that the amphipathic CTD of Cpx1/2 directly associates with lipid bilayers, with negatively charged lipids such as phosphatidylserine playing important roles in this interaction^22,25,33,80,82–84^. Consistent with these findings, we observed that Cpx2 directly interacted with protein-free, PS-containing liposomes in liposome co-flotation assays, and that this membrane binding was abolished upon deletion of the CTD (Fig. 4d).

Next, we examined whether Cpx2 directly interacts with GLUT4 exocytic SNAREs. While Cpx-SNARE interactions have been characterized in synaptic exocytosis, GLUT4 exocytic SNAREs differ from synaptic SNAREs in both sequence and physiological function^85,86^. In insulin-triggered GLUT4 exocytosis, the primary t-SNAREs are syntaxin-4 and SNAP-23, whereas the main cognate v-SNARE is VAMP2/synaptobrevin^69,85^. In a liposome co-flotation assay, Cpx2 bound to liposomes containing the trimeric GLUT4 exocytic SNARE complex composed of both the v- and t-SNAREs (Fig. 4d). The Cpx2 ΔCTD mutant, which does not bind to protein-free liposomes, still bound to these SNARE liposomes (Fig. 4d). By contrast, the Cpx2 ΔCTD mutant did not bind appreciably to liposomes containing the dimeric t-SNARE complex (syntaxin-4 and SNAP-23) (Fig. 4d), indicating Cpx2 selectively interacts with the trimeric SNARE complex formed by the v- and t-SNAREs. A previous study examined a hybrid SNARE complex of syntaxin-4 with synaptic SNAREs and observed no Cpx binding^87^. Using the native GLUT4 exocytic SNARE complex, our biochemical data demonstrate that Cpx directly binds this physiologically relevant SNARE assembly.

Together, these findings suggest that Cpx2 interacts with membranes via its CTD while engaging GLUT4 exocytic SNAREs through a distinct region of the protein.

### The Syt-independent function of Cpx2 requires an interaction between its CH and GLUT4 exocytic SNAREs

**Fig. 5:**
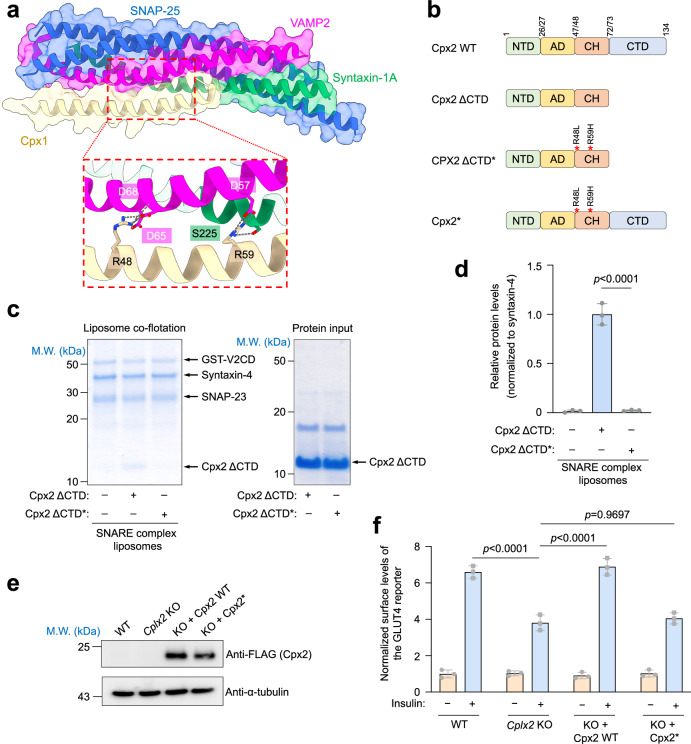
The Syt-independent function of Cpx2 requires direct interaction of its CH domain with the GLUT4 exocytic SNARE complex. **a** Crystal structure of rat Cpx1 (a.a. 32–72) bound to the synaptic SNARE complex (PDB: 1KIL). The inset highlights the interactions of residues R48 and R59 of Cpx1 with VAMP2 and syntaxin-1A. These residues occupy the same positions in human Cpx2 used in this study. The structural model was visualized using ChimeraX v1.9. **b** Diagrams of WT and mutant Cpx2 proteins used in (**c**) and (**e**). **c** Representative Coomassie blue-stained gels from three independent experiments showing the binding of Cpx2 to the GLUT4 exocytic SNARE complex in liposome co-flotation assays. SNARE complex liposomes were prepared as in Fig. 4d. Liposomes and associated proteins were isolated on a Nycodenz density gradient, and analyzed using SDS-PAGE and Coomassie blue staining. Since the CTD of Cpx2 binds to the membrane bilayer, Cpx2 ΔCTD was used in the assays to characterize SNARE interactions. **d** Quantification of protein levels from SDS-PAGE analysis. Densitometric quantification of protein levels was performed based on three independent experiments as described in (**c**). For normalization, the mean value of syntaxin-4 was set to 1, and all individual data points were normalized accordingly. Data are presented as mean ± SD of three biological replicates. Statistical significance was determined by one-way ANOVA followed by Šídák’s multiple comparisons test. **e** Representative immunoblots from three independent experiments showing the expression of the indicated proteins in adipocytes. **f** Normalized surface levels of GLUT4 reporters in the indicated adipocytes. The cells were either untreated or treated with 100 nM insulin for 30 min before surface levels of GLUT4 reporters were measured using flow cytometry. Data are presented as mean ± SD of three biological replicates. *p* values were calculated using one-way ANOVA and Tukey’s multiple comparison test.

Next, we investigated whether the Syt-independent function of Cpx2 requires direct interactions with the GLUT4 exocytic SNARE complex. Although a structure of Cpx2 bound to these SNAREs is not yet available, the crystal structure of Cpx1 bound to the synaptic SNARE complex has been solved^88^. In this structure, Cpx1 binds the SNARE complex in an anti-parallel orientation, with the CH making contacts with the v-SNARE VAMP2 and the t-SNARE syntaxin-1A (Fig. 5a)^88^. Two residues in the CH, R48 and R59, form critical interactions with these SNAREs and are required for complex formation^81,88^. Specifically, R48 forms hydrogen bonds with D65 and D68 of VAMP2, whereas R59 forms hydrogen bonds with D57 of VAMP2 and S225 of syntaxin-1A (Fig. 5a)^88^. These residues are evolutionarily conserved and occupy the same positions in Cpx2 (Fig. 5b).

Because SNARE complexes share a conserved four-helix bundle architecture and both synaptic and GLUT4 exocytic pathways use VAMP2 as the v-SNARE^7,12,86^, we hypothesized that R48 and R59 also mediate Cpx2 binding to the GLUT4 exocytic SNARE complex. To test this, we introduced R48L/R59H mutations and found that these substitutions abolished the ability of Cpx2 ΔCTD to bind the GLUT4 exocytic SNARE complex in liposome co-flotation assays (Fig. 5c, d). Thus, these conserved residues are essential for Cpx2 binding to the GLUT4 exocytic SNARE complex. We next expressed a full-length (FL) Cpx2 mutant bearing the R48L/R59H substitutions in *Cplx2* KO adipocytes at levels comparable to WT Cpx2 (Fig. 5e) and found that it failed to restore insulin-triggered GLUT4 exocytosis (Fig. 5f). These results demonstrate that the Syt-independent function of Cpx2 in regulated exocytosis requires a direct interaction between its CH and the SNARE complex.

### The Syt-independent function of Cpx2 in regulated exocytosis requires its CTD-mediated membrane remodeling

**Fig. 6:**
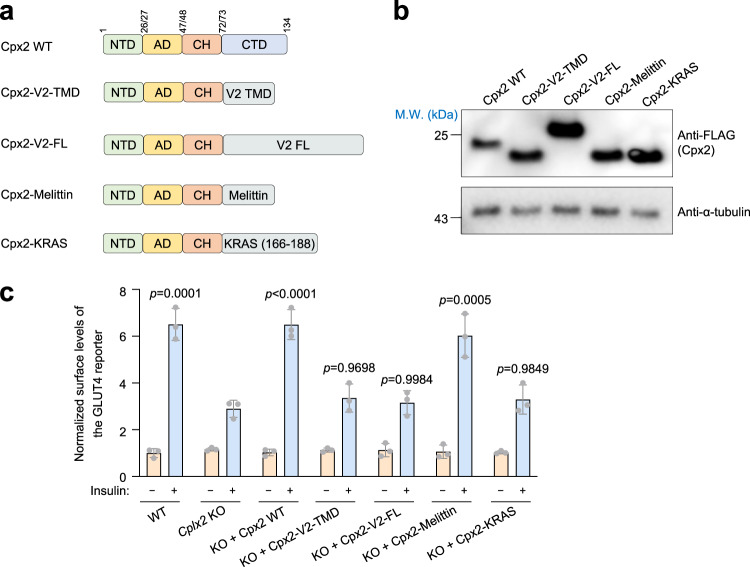
The Syt-independent function of Cpx2 depends on the membrane-remodeling activity of its CTD. **a** Diagrams of Cpx2 chimeras in which the CTD of Cpx2 was replaced with the TMD (a.a. 93-116) of mouse VAMP2 (V2), FL V2 (a.a. 1-116), melittin, or the lipid-conjugated region of human KRASB (a.a. 166-188). **b** Representative immunoblots from three independent experiments showing the expression of the indicated proteins in adipocytes. **c** Normalized surface levels of GLUT4 reporters in WT adipocytes, *Cplx2* KO adipocytes, and KO adipocytes expressing the indicated proteins. The cells were either untreated or treated with 100 nM insulin for 30 min before surface levels of GLUT4 reporters were measured using flow cytometry. Data are presented as mean ± SD of three biological replicates. Data from insulin-treated samples were compared to those from insulin-treated *Cplx2* KO adipocytes. *p* values were calculated using one-way ANOVA and Tukey’s multiple comparison test.

Finally, we sought to define the molecular mechanism by which the amphipathic, membrane-binding CTD contributes to the Syt-independent function of Cpx2. Deletion of the CTD abolished the ability of Cpx2 to regulate insulin-stimulated GLUT4 exocytosis, as well as its ability to enhance basal surface levels of GLUT4 in WT adipocytes (Fig. 4c and Supplementary Fig. 9). To test whether the CTD primarily mediates membrane association of Cpx2, we replaced it with the transmembrane domain (TMD) of VAMP2 or FL VAMP2 (Fig. 6a), a strategy known to reliably anchor Cpx to membranes near vesicle fusion sites^33^. However, neither of the Cpx2–VAMP2 chimeras supported insulin-triggered GLUT4 exocytosis when expressed in *Cplx2* KO adipocytes (Fig. 6b, c). Likewise, replacing the CTD with a fatty acid–conjugating sequence from KRAS, which targets proteins to the plasma membrane^89^, also failed to restore GLUT4 exocytosis (Fig. 6b, c). To further explore the role of the CTD, we replaced it with the exocytic Rab GTPases Rab3A or Rab10. This Rab-fusion strategy has been shown to effectively localize Cpx to exocytic vesicles^80^. We observed that neither of the Cpx–Rab chimeras rescued GLUT4 exocytosis when expressed in *Cplx2* KO adipocytes (Supplementary Fig. 10). These data indicate that the CTD of Cpx2 performs a function beyond simple membrane association.

We next replaced the CTD with melittin, a 26-residue amphipathic peptide from bee venom (Fig. 6a)^22,90^. In reconstituted assays, melittin mimics the Cpx CTD in membrane binding and remodeling^22^, but whether it can functionally substitute for the CTD in a physiological cellular context was unknown. Remarkably, the Cpx2-melittin chimera fully restored insulin-triggered GLUT4 exocytosis when expressed in *Cplx2* KO adipocytes (Fig. 6b, c and Supplementary Fig. 10). Mutations of the SNARE-binding sites in the CH domain abolished the ability of the Cpx2–melittin chimera to support insulin-stimulated GLUT4 exocytosis (Supplementary Fig. 10), suggesting that the chimera regulates exocytosis through the same SNARE-dependent mechanism as WT Cpx2. Because melittin shares no sequence similarity with the Cpx2 CTD, this result supports a model that CTD remodels membrane bilayers during exocytic vesicle fusion.

Altogether, our findings demonstrate that Cpx2 integrates membrane remodeling and SNARE binding as dual requirements for its Syt-independent function in regulated exocytosis.

## Discussion

This study uncovered a Syt-independent function of Cpx in a regulated exocytic pathway that naturally lacks Syt – insulin-triggered GLUT4 exocytosis. In this pathway, Cpx2 (referred to hereafter as Cpx) accelerates exocytosis during insulin stimulation without affecting basal fusion, a behavior distinct from its well-characterized, Syt-dependent role in Ca²⁺-triggered exocytosis. The Syt-independent stimulatory activity of Cpx relies on two key molecular interactions: binding to the SNARE complex through its CH domain and membrane engagement via its amphipathic CTD.

Through its interaction with SNAREs, the CH is expected to position Cpx at sites of membrane fusion. In addition to this anchoring role, the CH may also play a more active part by facilitating SNARE zippering, consistent with evidence implicating Cpx in vesicle docking^91^. The CTD of Cpx has been proposed to serve multiple mechanistically distinct functions, including membrane association, vesicle targeting, and direct modulation of membrane structure^25,33,80^. Our findings suggest that in a Syt-independent exocytic pathway, the CTD contributes more than simple membrane association; rather, its biochemical properties enable it to promote bilayer remodeling. Because membrane fusion involves extensive lipid disruption and rearrangement – creating a high-energy barrier that must be overcome by specialized membrane fusion proteins^7,92,93^—the amphipathic CTD likely promotes fusion by inserting into the bilayer to locally perturb lipid packing and facilitate lipid reorganization. This model is consistent with in vitro observations that the Cpx CTD stabilizes nascent fusion pores and stimulates liposome fusion^22,83^. However, we cannot rule out the possibility that the CTD primarily serves to concentrate Cpx at specific membrane locations, such as high-curvature regions.

**Fig. 7:**
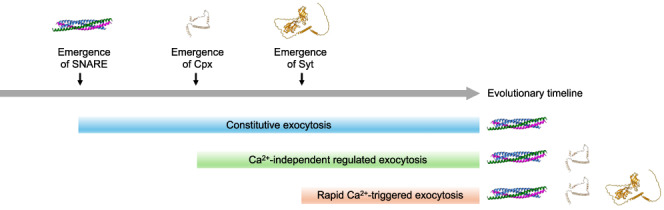
Model illustrating the evolutionary emergence of Syt-independent and Syt-dependent functions of Cpx in regulated exocytosis. SNARE-mediated constitutive exocytosis, which operates without Cpx or Syt, arose first. This was followed by the evolution of Ca²⁺-independent regulated exocytosis that required Cpx. With the subsequent emergence of Syt, rapid Ca²⁺-triggered exocytosis evolved, in which Syt functions together with Cpx to achieve precise Ca²⁺ regulation, while the ancestral Syt-independent role of Cpx was retained. The structural model of the SNARE complex is based on a crystal structure (PDB: 1SFC)^116^, while the models of Cpx and Syt are based on the predicted structures of Cpx2 and Syt1 from the AlphaFold Protein Structure Database (#AF-Q6PUV4-F1 and #AF-P21579-F1, respectively). The evolutionary timeline is not to scale. For clarity, other SNARE-binding regulators are not shown.

These findings reveal that the intrinsic activity of Cpx is solely exocytosis-accelerating, with no clamping function at any stage of the exocytic process. Because core functions of vesicle fusion regulators are conserved throughout evolution^6,7,94,95^, the stimulatory role of Cpx identified in this mammalian pathway likely reflects its primordial function in exocytosis, predating the emergence of Syt. During the evolution of the endomembrane system, SNARE proteins arose early to mediate cargo delivery to the plasma membrane by constitutive exocytosis, as well as cargo transport between endomembrane organelles (Fig. 7). However, SNAREs and other early-evolved factors alone were insufficient to support the rapid, stimulus-dependent release of cargo required for adaptive responses to environmental changes. Cpx therefore evolved to accelerate stimulus-triggered exocytosis, enabling faster cargo mobilization. With the later appearance of the Ca²⁺ sensor Syt, Ca²⁺-triggered exocytic pathways emerged, during which Cpx acquired an additional role–acting together with Syt to clamp vesicle fusion before Ca²⁺ influx, thereby permitting tight Ca²⁺ regulation and ultrafast exocytic responses (Fig. 7). Although Ca²⁺-triggered exocytosis is now the dominant form of regulated exocytosis in metazoans, the ancestral Syt-independent function of Cpx has been retained.

While the Syt-independent stimulatory activity uncovered in this work likely reflects a primordial role of Cpx, this function may have been modified during evolution to meet species-specific demands. For example, the subsequent emergence of a clamping role alongside Syt may have required adjustments in domain organization and in the functional contributions of individual regions, while still preserving the underlying stimulatory activity in exocytosis. Consistent with this idea, while the CH domain is the most conserved region of Cpx, other domains show substantial divergence^25,38^. Notably, Cpx proteins from some organisms lack the amphipathic CTD—a region essential for membrane remodeling in mammalian Cpx1/2^22,25^. We therefore postulate that other sequences within these Cpxs may fulfill analogous functions in membrane binding. Supporting this possibility, the NTD of Cpx from certain species has been shown to exhibit membrane-binding activity^84,96,97^. In addition, some Cpxs, such as mammalian Cpx3 and Cpx4, are associated with membranes through a C-terminal CaaX motif that undergoes lipid modification. Comparative analyses of these diverse Cpx proteins will be important for identifying both conserved features and points of evolutionary divergence.

Our findings also provide insight into the mechanism of Cpx in Ca²⁺-triggered synaptic exocytosis and may help reconcile long-standing controversies in the field. We propose that, in addition to its well-established Syt-dependent clamping activity, Cpx also exerts a stimulatory, Syt-independent role in synaptic exocytosis after clamp release—similar to the activity identified in this study. This dual functionality aligns with previous models proposing both inhibitory and facilitatory roles for Cpx in synaptic neurotransmitter release^41,98^. Although clamping is a universal function of Cpx in Ca²⁺-triggered synaptic exocytosis, the extent to which its Syt-independent stimulatory activity is engaged likely varies across species and experimental systems. Consequently, the relative balance between these two activities may underlie the diversity of Cpx loss-of-function phenotypes observed across systems. For instance, cultured mammalian neurons lacking Cpx often show reduced spontaneous neurotransmitter release^37,99^, which may reflect a prominent stimulatory role of Cpx once the clamp is lifted by Ca²⁺. In this context, Cpx loss eliminates both clamping and stimulation, and because efficient fusion depends on the latter, spontaneous release decreases. In contrast, in other experiments with cultured mammalian neurons, silencing Cpx expression increases spontaneous release^100,101^, likely because the post-clamp stimulatory activity is less dominant or is compensated by other facilitatory factors. Likewise, a weaker Syt-independent contribution may explain why Cpx deletion elevates spontaneous release in nematodes and fruit flies^32,33,35,102^.

A key future direction is to further define the molecular basis of the Syt-independent function of Cpx. In particular, it will be important to clarify how the NTD and AD contribute to this activity. Although these domains are less critical than the CH and CTD, they nonetheless exert positive modulatory effects on fusion in the GLUT4 exocytic pathway. These effects may arise because the NTD and AH support the optimal function of the CH and CTD, or because they play more active roles through independent interactions with SNAREs or membrane bilayers. Another critical question is how the domain requirements for the Syt-independent stimulatory activity differ from those for clamping. Notably, the same regions that support the stimulatory activity are also implicated in clamping during Ca²⁺-triggered exocytosis^33,82,103^, suggesting that they engage similar elements of the fusion machinery while executing distinct functional outputs. Such overlap is not unexpected given the compact size of Cpx, although the precise configuration of these interactions likely differs between the two modes. Addressing these questions will require integrating biochemical reconstitution with targeted genetic perturbation across multiple experimental systems. Finally, it will also be important to determine whether and how Cpx functions alongside other C2-domain proteins such as Doc2. Although classic Syt-dependent Ca²⁺-triggered exocytosis does not occur in adipocytes, certain adipose exocytic events may exhibit partial Ca²⁺ dependence^104^. While Cpx is not known to physically interact with Doc2, and Doc2 does not clamp SNARE zippering as Syt does, the two proteins may nevertheless act in a coordinated or complementary manner to regulate exocytosis.

## Methods

### Cell culture

Immortalized mouse preadipocytes (derived from mouse inguinal white adipocyte tissues, a gift from Dr. Shingo Kajimura, Harvard University) and HEK 293 T cells were cultured in Dulbecco’s Modified Eagle Medium (DMEM) supplemented with 10% Fetal Bovine Essence (FBE, VWR, #10803-034) and penicillin/streptomycin (Thermo Fisher Scientific, #15140122). All cell lines were maintained in a humidified 37 °C incubator with 5% CO_2_. Mouse preadipocytes were differentiated into mature adipocytes using an established procedure^65,105^. Preadipocytes were grown to ~95% confluence before a differentiation cocktail was added at the following final concentrations: 5 µg/mL insulin (Sigma-Aldrich, #I0516), 1 nM Triiodo-L-thyronine (T3, Sigma-Aldrich, #T2877), 125 µM indomethacin (Sigma-Aldrich, #I7378), 5 µM dexamethasone (Sigma-Aldrich, #D1756), and 0.5 mM 3-isobutyl-1-methylxanthine (IBMX, Sigma-Aldrich, #I5879). After 2 days, the cells were switched to DMEM supplemented with 10% FBE, 5 µg/mL insulin, and 1 nM T3. After another 2 days, cell culture media were replaced with fresh DMEM media supplemented with 10% FBE and 1 nM T3. Adipocytes were analyzed 6 days after switching to the differentiation cocktail.

### Gene KO using CRISPR-Cas9

Each candidate gene was simultaneously targeted by two gRNAs. Oligonucleotides of the two gRNAs were subcloned into the pLenti-CRISPR-V2 vector (Addgene, #52961) or the pLentiGuide-Hygro vector, respectively^105^. CRISPR plasmids were transfected into HEK 293 T cells together with pAdVAntage (Promega, #E1711), pCMV-VSV-G (Addgene, #8454), and psPAX2 (Addgene, #12260) using a previously established procedure^106^. HEK 293 T cell culture media containing lentiviral particles were harvested daily for 4 days and centrifuged at 112,400 × *g* for 1.5 h using a Beckman SW28 rotor. Viral pellets were resuspended in PBS and used to infect target cells. After lentiviral infection, cells were sequentially selected using 1 µg/mL puromycin (Sigma-Aldrich, #P8833) and 500 µg/mL hygromycin B (Thermo Fisher Scientific, #10687010). Oligonucleotide sequences of gRNAs targeting the mouse *Sytl2* gene are 5’-CTGGGACATATCAATCACAC-3’ and 5’-CTCTGATGATGTCAGCACCG -3’. Oligonucleotide sequences of gRNAs targeting the mouse *Cplx2* gene are 5’-GTCATGAAGCAAGCCCTCGG -3’ and 5’- GGCAGAGGAGAAGGCAGCCC-3’. Oligonucleotide sequences of gRNAs targeting the mouse *Cplx1* gene are 5’-GAGGAACCAAGCCATCACCA-3’ and 5’- GTATGGCATCAAGAAGAAGG-3’.

### Gene expression in mammalian cells

The human *CPLX1*, *CPLX2*, *CPLX3*, and *CPLX4* genes were subcloned into the NheI and SalI sites of the SHC003BSD-GFPD vector (Addgene, #133301) with a 3×FLAG sequence at either the 5′ or 3′ ends. Cpx2 mutants were generated using a site-directed mutagenesis kit (Agilent, #210518). DNA fragments encoding Cpx2 chimeras were ordered from Integrated DNA Technologies and subcloned into the NheI and SalI sites of the SHC003BSD-GFPD vector. In these chimeras, the CTD of Cpx2 was replaced with the TMD (a.a. 93-116) of mouse VAMP2 (V2), full-length (FL) V2 (a.a. 1-116), melittin (sequence: GIGAILKVLATGLPTLISWIKNKRKQ), the lipid-conjugated region of human KRASB (a.a. 166-188; sequence: HKEKMSKDGKKKKKKSKTKCVIM), mouse FL Rab3A (a.a. 1-220), or mouse FL Rab10 (a.a. 1-200). The plasmids were transfected into HEK 293 T cells to produce lentiviral particles using the same procedure as for CRISPR lentiviral production. Lentiviruses were used to infect target cells, followed by selection with blasticidin (10 μg/mL; Thermo Fisher Scientific, #BP2647).

### Flow cytometry analysis

Adipocytes stably expressing the GLUT4 reporter were washed three times with KRH buffer and starved in KRH buffer for 2 h. Cells were then incubated in KRH buffer containing insulin for the indicated durations, chilled on ice, and blocked with 5% FBE in KRH buffer at 4 °C. Surface GLUT4 reporters were stained using anti-HA antibodies followed by APC-conjugated secondary antibodies. Cells were dissociated using Accutase and analyzed on a CyAN ADP analyzer (Beckman Coulter) or a MACSQuant Analyzer (Miltenyi Biotec). Data were analyzed with FlowJo software (FlowJo, LLC) from biological triplicates. Background fluorescence intensities were subtracted using GFP-negative cells to define GFP autofluorescence and unstained adipocytes to calibrate the APC channel baseline before calculating corrected mean or median intensities. Normalized surface GLUT4 reporter levels were calculated by dividing mean APC fluorescence by mean GFP fluorescence, the latter reflecting total GLUT4 reporter levels.

### Lipid droplet staining and analysis

Preadipocytes were grown in clear-bottom 96-well plates. After differentiation, adipocytes were fixed with 4% PFA and stained with Oil Red O (Sigma-Aldrich, #O0625). Images of stained lipid droplets were captured using a bright-field microscope. In parallel, stained cells were air-dried at room temperature for 2 h, after which methanol was added to solubilize Oil Red O. Absorbance of the supernatant was measured at 520 nm on a plate reader. The absorbance of KO cells was normalized to WT cells. Experiments were performed in biological triplicate.

### Immunostaining and imaging

To visualize Cpx2, cells grown on coverslips were fixed using 4% PFA and permeabilized in PBS containing 5% FBE and 0.2% saponin. Endogenous Cpx2 was labeled using anti-Cpx1/2 primary antibodies (Cell Signaling Technology, #28070, RRID: AB_2798954) and Alexa Fluor 568-conjugated secondary antibodies (Thermo Fisher Scientific, #A11004, RRID: AB_2534072). To visualize surface GLUT4 reporters, preadipocytes cultured in four-chamber glass bottom dishes were differentiated into mature adipocytes, fixed with 4% paraformaldehyde, and blocked with 2% bovine serum albumin (BSA) without permeabilization. Surface reporters were stained with anti-HA antibodies and Alexa Fluor 568-conjugated secondary antibodies (Thermo Fisher Scientific, #A11004, RRID: AB_2534072). Nuclei were stained with Hoechst 33342 (Sigma-Aldrich, #D9642).

### Mass spectrometry

For whole-cell proteomic analysis, adipocytes and CAD cells were collected directly into lysis buffer containing 100 mM triethylammonium bicarbonate (Millipore-Sigma, #18597), 5 mM tris(2-carboxyethyl)phosphine, 20 mM chloroacetamide (Millipore-Sigma, #C0267), and 1% SDS. Protein samples from three biological replicates were processed for mass spectrometry using the protein aggregation capture method and labeled with the TMTsixplex Isobaric Label Reagent Set (Thermo Fisher Scientific, #90061)^107^. Briefly, proteins were chemically aggregated onto magnetic microparticles by adding acetonitrile to a final concentration of 70% (vol/vol). The microparticles were washed with 95% acetonitrile and 70% ethanol to remove detergents and lipids. Proteins were digested on-bead with sequencing-grade trypsin at a 1:100 enzyme-to-substrate ratio in 50 mM TEAB for 16 h at 37 °C. Peptides were recovered from the supernatant, acidified with formic acid, and adjusted to a volume of 100 μL with 100 mM TEAB for chemical labeling. Amine-reactive tagging was carried out by adding 41 μL of the TMTsixplex reagent, freshly dissolved in anhydrous acetonitrile, to each sample and incubating for 1 h at room temperature. The reactions were subsequently quenched by the addition of 8 μL of 5% hydroxylamine for 15 min at room temperature before the channels were pooled.

Tryptic peptides were separated using a Pierce high-pH reversed-phase spin column with an 18-step acetonitrile gradient. The first fraction was eluted with 4% acetonitrile, fractions 2–17 were eluted with successive 1% acetonitrile increments, and the final fraction was eluted with 50% acetonitrile. Fractions were dried by vacuum centrifugation and analyzed on a Thermo Ultimate 3000 RSLCnano system operated in direct-injection mode. One-third of each high-pH fraction, corresponding to 5 μL, was subjected to UPLC-MS/MS^107^. Peptides were loaded onto a Waters nanoACQUITY UPLC BEH C18 column, 130 Å, 1.7 μm, 75 μm × 250 mm, equilibrated in 0.1% formic acid, 3% acetonitrile, and water. Mobile phases consisted of 0.1% formic acid in water, phase A, and 0.1% formic acid in acetonitrile, phase B. Peptides were separated at a flow rate of 0.3 µL/min using a gradient from 3% to 8% phase B over 5 min, followed by 8% to 35% phase B from 5 to 123 min.

Mass spectrometric acquisition was performed on an LTQ Orbitrap Velos mass spectrometer. Precursor ions were scanned over 300–1800 m/z at a resolution of 60,000 with a target of 1 × 10⁶ ions. The 10 most abundant precursor ions were selected for MS/MS with dynamic exclusion set to 180 s, an exclusion width of 10 ppm, repeat count of 1, and repeat duration of 30 s. Singly charged ions and ions with unassigned charge states were excluded from fragmentation. Maximum ion injection times were 500 ms for Fourier transform scans, with one microscan, and 250 ms for LTQ scans. The automatic gain control target for MS/MS was 1 × 10⁴. Fragmentation was performed using a normalized collision energy of 35%, activation Q of 0.25, and activation time of 10 ms.

Raw mass spectrometry files were analyzed with MaxQuant/Andromeda version 1.6.2.10 and searched against the UniProt mouse protein sequence database^108^. Database searches specified trypsin digestion with up to two missed cleavages. Carbamidomethylation of cysteine was set as a fixed modification, whereas protein N-terminal acetylation and methionine oxidation were included as variable modifications. Precursor mass tolerances were 20 ppm for the first search and 4.5 ppm for the main search; the MS/MS mass tolerance for ion trap spectra was 0.5 Da. MaxQuant/Andromeda analysis used the eight most intense MS/MS peaks per 100 Da, required a minimum peptide length of seven amino acids, and applied a false discovery rate of 1% at both the peptide and protein levels.

### Immunoblotting

To prepare whole cell lysates for immunoblotting, cells grown in 24-well plates were lysed in an SDS protein sample buffer (80 mM Tris, pH 6.8, 2% SDS, 10% glycerol, 0.0006% Bromophenol blue, and 0.1 M DTT). Proteins in cell lysates were resolved on 8% Bis-Tris SDS-PAGE, transferred to PVDF membranes, and probed using antibodies. Primary antibodies used in immunoblotting included anti-Cpx1/2 antibodies (Cell Signaling Technology, #28070, RRID: AB_2798954), anti-phospho-Akt (Ser^473^) antibodies (Cell Signaling Technology, #9271, RRID: AB_2716452), anti-Akt antibodies (Cell Signaling Technology, #9272, RRID: AB_329827), anti-peroxisome proliferator-activated receptor γ (Santa Cruz Biotechnology, #sc-7273, RRID: AB_628115), anti-AS160 antibodies (Cell Signaling Technology, #2670, RRID: AB_2199375), anti-phospho-AS160 (Thr^642^) antibodies (Cell Signaling Technology, #8881, RRID: AB_2651042), anti-Syt1 (clone m48), anti-Syt7 (Santa Cruz Biotechnology, #sc-293343) and anti-α-tubulin (DSHB, clone: #12G10, RRID: AB_1210456). Secondary antibodies used in immunoblotting included horseradish peroxidase (HRP)-conjugated anti-rabbit antibodies (Sigma-Aldrich, #A6154, RRID: AB_258284), and HRP-conjugated anti-mouse antibodies (Sigma-Aldrich, #A6782, RRID: AB_258315). FLAG-tagged proteins were directly detected using HRP-conjugated anti-FLAG M2 antibodies (Sigma-Aldrich, #A8592, RRID: AB_439702).

### Quantitative reverse transcription PCR (qRT-PCR)

Total RNAs were isolated using a RNeasy Mini Kit (Qiagen, #74104). After treatment with ezDNAse (Thermo Fisher Scientific, #11766051), the first-strand complementary DNA was synthesized using a SuperScript IV kit (Thermo Fisher Scientific, #18091050). Gene expression was quantified using qRT-PCR on an Applied Biosystems™ 7500 Fast Real-Time PCR System using SsoAdvanced Universal SYBR Green Supermix (Bio-Rad, #172-5272) with gene-specific primer sets. Cycle threshold values of *Sytl2* were normalized to those of *Gapdh*, which was used as a reference gene. PCR primers for *Sytl2* were: 5’-GCCCAAGTTCCAGTTTGGTG-3’ (forward) and 5’-ACACAGCTGGAGTCCTCGTA-3’ (reverse). PCR primers for *Gapdh* were: 5’-AGGTCGGTGTGAACGGATTTG-3’ (forward) and 5’-TGTAGACCATGTAGTTGAGGTCA-3’ (reverse).

### Recombinant protein expression

Recombinant proteins were expressed and purified as previously described^85,86,109^. The GLUT4 exocytic t-SNARE complex was composed of untagged rat syntaxin-4 and mouse His_6_-tagged SNAP-23^85,86^. The cytoplasmic domain of mouse VAMP2 (GST-VAMP2 CD) was purified by affinity chromatography. Human *CPLX2* was subcloned into the BamHI and XhoI sites of the pET-SUMO expression vector^110,111^.

Recombinant proteins were expressed in BL21 (DE3) *E. coli* (Stratagene, #230132). When the OD_600_ of *E. coli* cultured in 2×YT media reached ~0.6, 1 mM isopropyl β-D-1-thiogalactopyranoside (IPTG, GoldBio, #I2481C100) was added to induce protein expression. After 3 h of incubation at 37 °C, cells were harvested and lysed. After centrifugation, proteins were isolated using glutathione beads (Thermo Fisher Scientific, #PI16101) or nickel beads (Thermo Fisher Scientific, #PI-88222). Proteins were resolved on 8% Bis-Tris SDS-PAGE and detected using Coomassie blue staining.

### Liposome co-flotation assays

Liposome preparation and co-flotation assays were conducted as previously described^85^. All lipids were obtained from Avanti Polar Lipids Inc. To prepare t-SNARE liposomes, 1-palmitoyl-2-oleoyl-sn-glycero-3-phosphocholine (POPC), 1-palmitoyl-2-oleoyl-sn-glycero-3-phosphoethanolamine (POPE), 1-palmitoyl-2-oleoyl-sn-glycero-3-phosphoserine (POPS), and cholesterol were mixed in a molar ratio of 60:20:10:10. Proteoliposomes were formed by adding detergent-solubilized t-SNAREs to dried lipids followed by detergent dilution. Detergent was removed by overnight dialysis in Novagen dialysis tubes against reconstitution buffer (25 mM HEPES [pH 7.4], 100 mM KCl, 10% glycerol, and 1 mM DTT). The protein:lipid ratio was 1:500 for t-SNARE liposomes. Liposomes were isolated on a Nycodenz density gradient. To prepare liposomes containing the SNARE complex, t-SNARE liposomes were incubated with 5 μM GST-V2CD (the cytosolic domain of VAMP2) for 1 h at 4 °C before isolation on a Nycodenz density gradient.

The interactions of Cpx2 with liposomes were measured using a liposome co-flotation assay. Cpx2 was incubated with liposomes at 4 °C with gentle agitation for 1 h. An equal volume of 80% Nycodenz (w/v) in reconstitution buffer was then added, and the mixture was transferred to centrifuge tubes. Samples were overlaid with 200 μL each of 35% and 30% Nycodenz, followed by 20 μL reconstitution buffer on top, and centrifuged at 327,710 × *g* for 4 h in a Beckman SW55 rotor. Liposomes were collected from the 0/30% Nycodenz interface (2 × 20 μL) and analyzed by SDS-PAGE.

### Quantification and statistics

In this study, band intensities of immunoblots were quantified using ImageJ (v2.14) and normalized to α-tubulin bands. Data normalization was conducted by setting the mean value of WT or KO data points as 100% or 1, and all data points were normalized to that mean value. Additional information is included in the figure legends. Student’s *t*-test was used for comparisons between two groups, while ANOVA was applied for analyses involving more than two groups, using GraphPad Prism (v9.0). Error bars indicate SD.

### Reporting summary

Further information on research design is available in the Nature Portfolio Reporting Summary linked to this article.

## Supplementary information

Supplementary Information

Descriptions of Additional Supplementary Files

Supplementary Data 1

Supplementary Data 2

Reporting summary

Transparent Peer Review file

## Source data

Source Data

## Data Availability

Data supporting the findings of this study are included in the main article and Supplementary Information. Raw mass spectrometry data have been deposited in iProX (www.iProX.org) under accession number IPX0017612000. Source data are provided with this paper.
