# Supplementary material for: A primordial synaptotagmin-independent function of complexin in regulated exocytosis
Source: Nat Commun. 2026 Jun 27;17:8035. doi: 10.1038/s41467-026-74947-4 (PMC13454271; doi:10.1038/s41467-026-74947-4)
Supplement: Supplementary file 1 [file 41467_2026_74947_MOESM1_ESM.pdf]

Supplementary Information

A primordial synaptotagmin-independent function of complexin in regulated exocytosis

Xu Chen[1†#], Chun Wan[1#], Jingyi Wu[1], Yan Ouyang[1], Harrison Puscher[1&],
Haijia Yu[2], and Jingshi Shen[1*]

[1]Department of Molecular, Cellular and Developmental Biology, University of Colorado, Boulder, CO 80309, USA.
[2]Jiangsu Key Laboratory for Molecular and Medical Biotechnology, College of Life Sciences, Nanjing Normal University, Nanjing, 210023, China.
[†]Present address: Department of Food Science and Nutrition, Hong Kong Polytechnic University, Hong Kong, China.
[&]Present address: Department of Biological Sciences, University of Southern California, Los Angeles, CA 90089, USA.
[#]These authors contributed equally to this work.

[*]Correspondence: jingshi.shen@colorado.edu (J.S.)

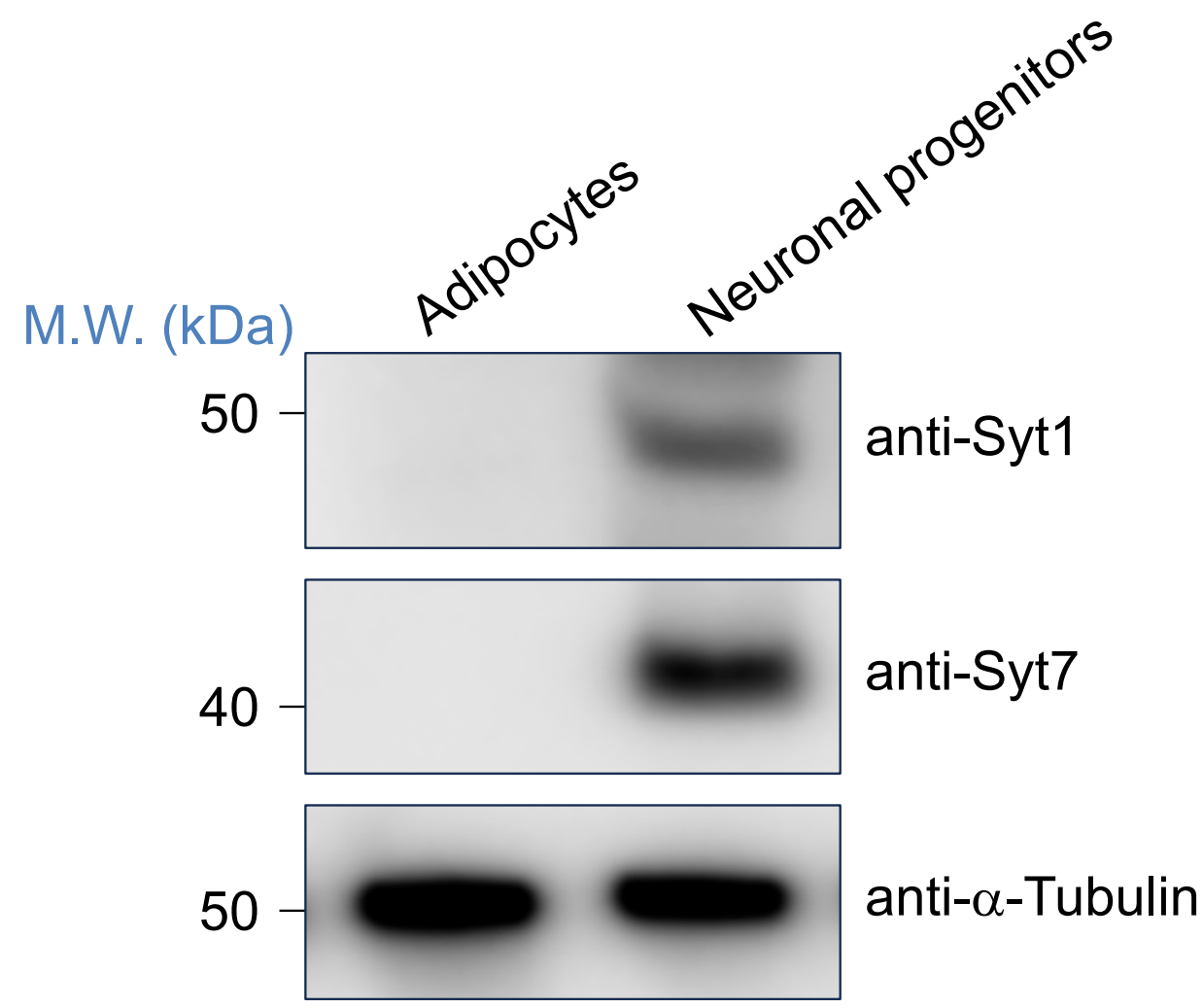

Supplementary Figure 1. Absence of synaptotagmin (Syt) expression in mouse adipocytes. Representative immunoblots from three independent experiments are shown to demonstrate that the exocytic Syts Syt1 and Syt7 are expressed in the Cath.a-differentiated (CAD) neuronal progenitor cell line but not in mouse adipocytes. Mouse adipocytes used in this study were differentiated *in vitro* from a preadipocyte cell line. Related to Figure 1.

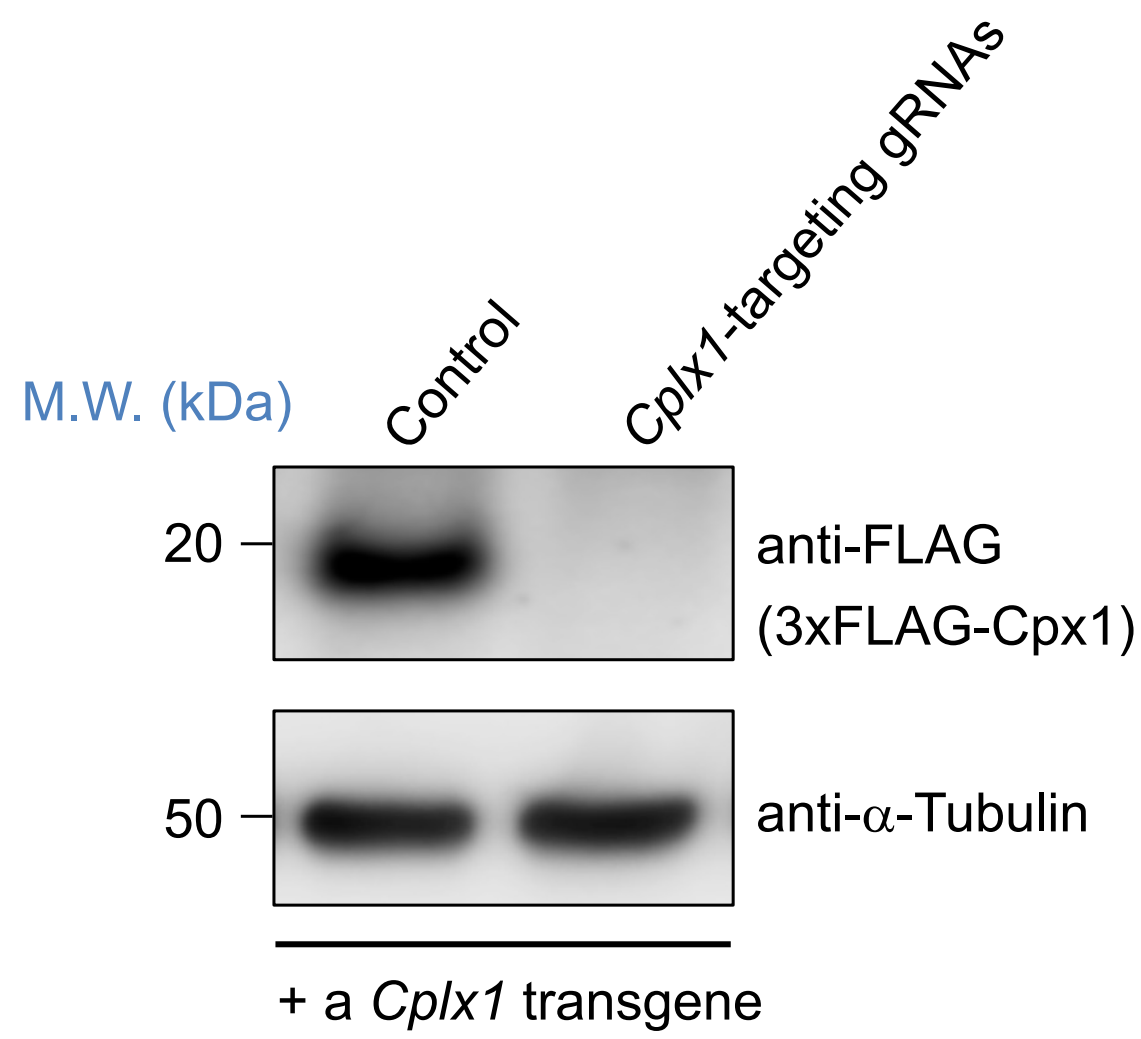

Supplementary Figure 2. Validation of *Cplx1* knockout (KO) efficiency in preadipocytes. Because Cpx1 is not endogenously expressed in preadipocytes or adipocytes, a transgene encoding 3×FLAG-tagged Cpx1 was introduced into wild-type (WT) preadipocytes. KO of the *Cplx1* gene was confirmed by loss of transgene expression, validating the efficiency of the CRISPR strategy and gRNAs used to target *Cplx1*. Representative immunoblots from three independent experiments are shown. Related to Figure 2.

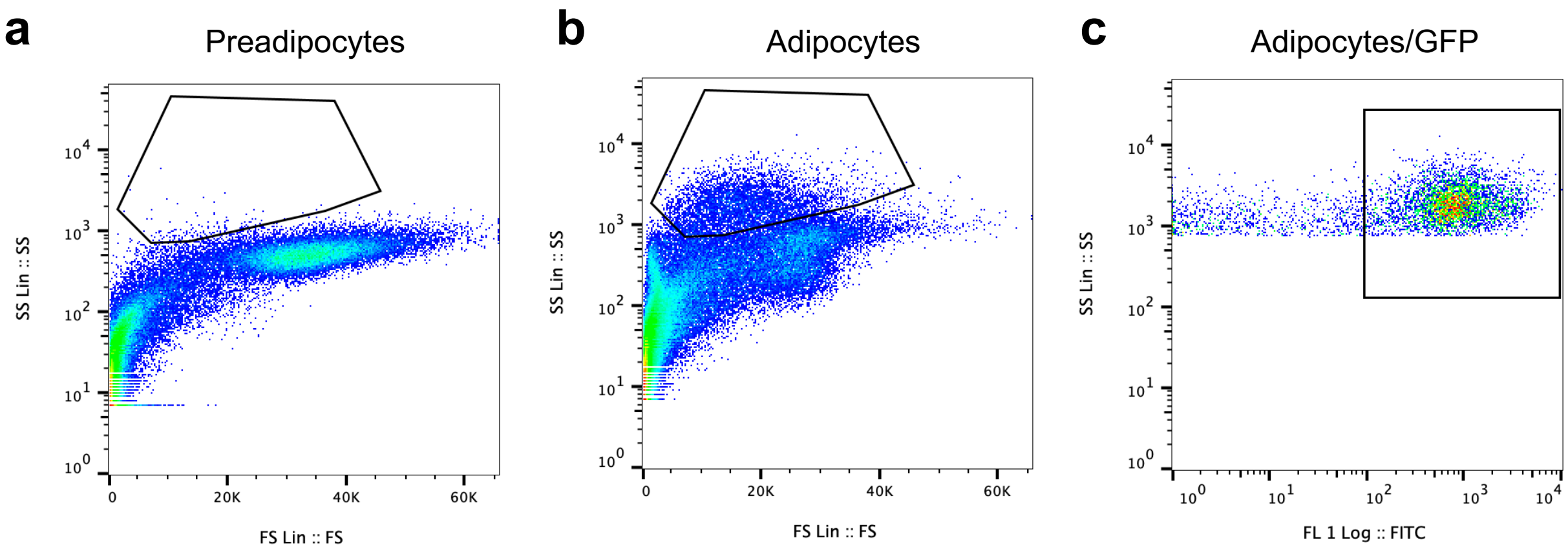

Supplementary Figure 3. Flow-cytometry gating strategy in adipocytes. Representative plots are shown to illustrate the sequential gating hierarchy used to identify mature adipocytes expressing the HA-GLUT4-GFP reporter. **(a)** Total events were first gated to identify the cell population and exclude debris. **(b)** A distinct shift in FSC/SSC (forward scatter/side scatter) compared to preadipocytes was used to define the adipocyte population. **(c)** The GFP-positive adipocyte population was then identified within this gate and used for downstream data analysis. Related to Figure 2.

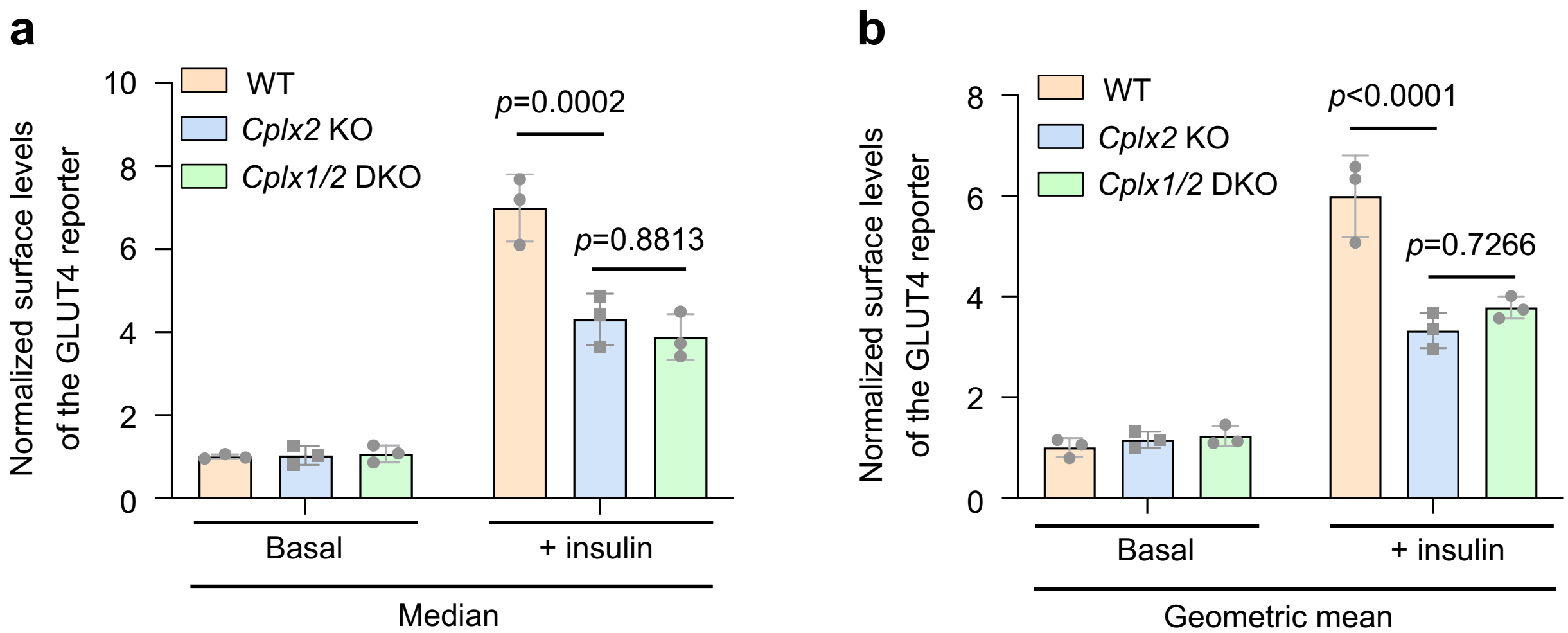

Supplementary Figure 4. Comparison of statistical measures for GLUT4 exocytosis quantification. The exocytosis of the HA-GLUT4-GFP reporter in this study was primarily quantified using mean fluorescence intensity. To account for the logarithmic distribution of flow cytometry data, representative datasets were additionally analyzed using the median (a) and geometric mean (b) of fluorescence intensities. These alternative analyses yielded conclusions consistent with those obtained using mean values. Data were normalized as in Figure 2c. Error bars indicate SD, n = 3. *P* values were calculated using two-way ANOVA with Tukey's multiple comparison test. Related to Figure 2.

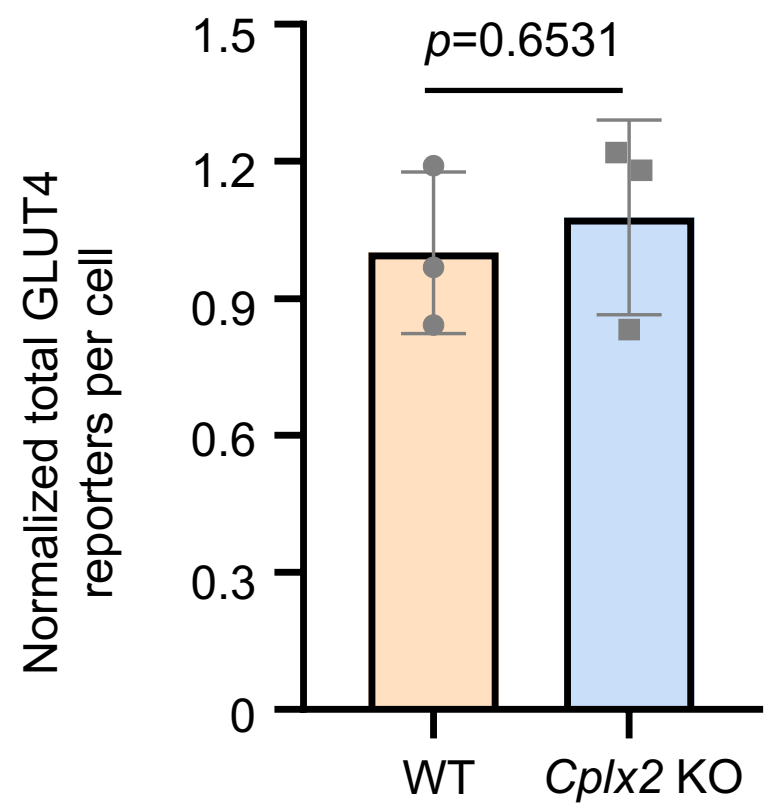

Supplementary Figure 5. Normalized total GLUT4 reporters in WT and *Cplx2* knockout (KO) mouse adipocytes. The HA-GLUT4-GFP reporter was quantified based on GFP fluorescence using flow cytometry. Data from KO cells were normalized to WT cells. Error bars indicate SD, n = 3. *P* values were calculated using two-sided Student's t-test. Related to Figure 2.

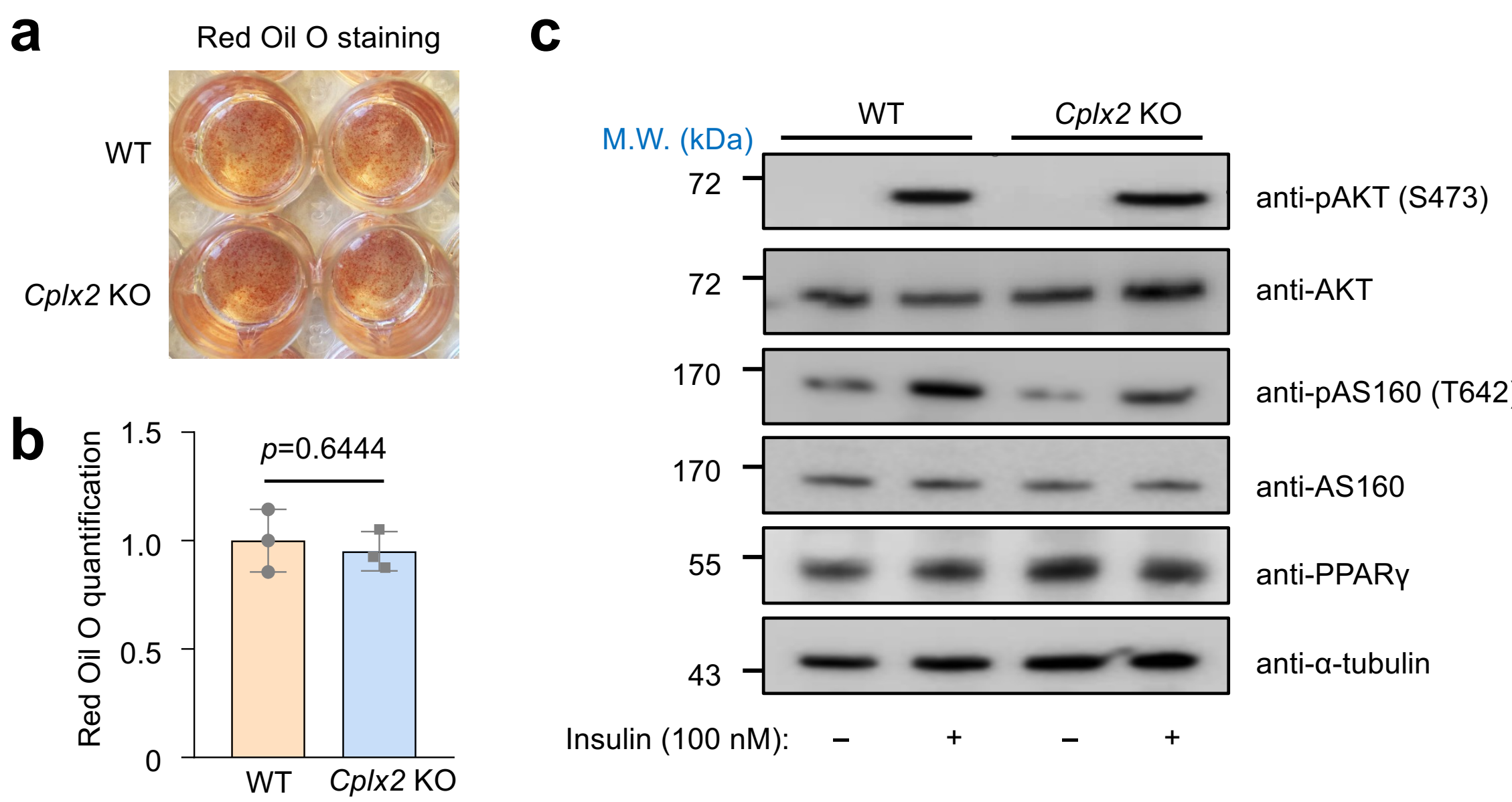

Supplementary Figure 6. Normal differentiation and insulin signaling in *Cplx2* KO adipocytes. (a) Representative images from three independent experiments showing Oil Red O staining of lipid droplets in WT and *Cplx2* KO mouse adipocytes. **(b)** Quantification of Oil Red O staining in WT and *Cplx2* KO adipocytes. Stained cells were solubilized and absorbance at 510 nm was measured on a plate reader. Data from KO cells were normalized to WT cells and are presented as mean ± SD, n = 3. *P* values were calculated using two-sided Student's t-test. **(c)** Representative immunoblots from three independent experiments showing expression of the indicated proteins in WT and *Cplx2* KO adipocytes, either untreated or treated with 100 nM insulin for 30 minutes. Related to Figure 2.

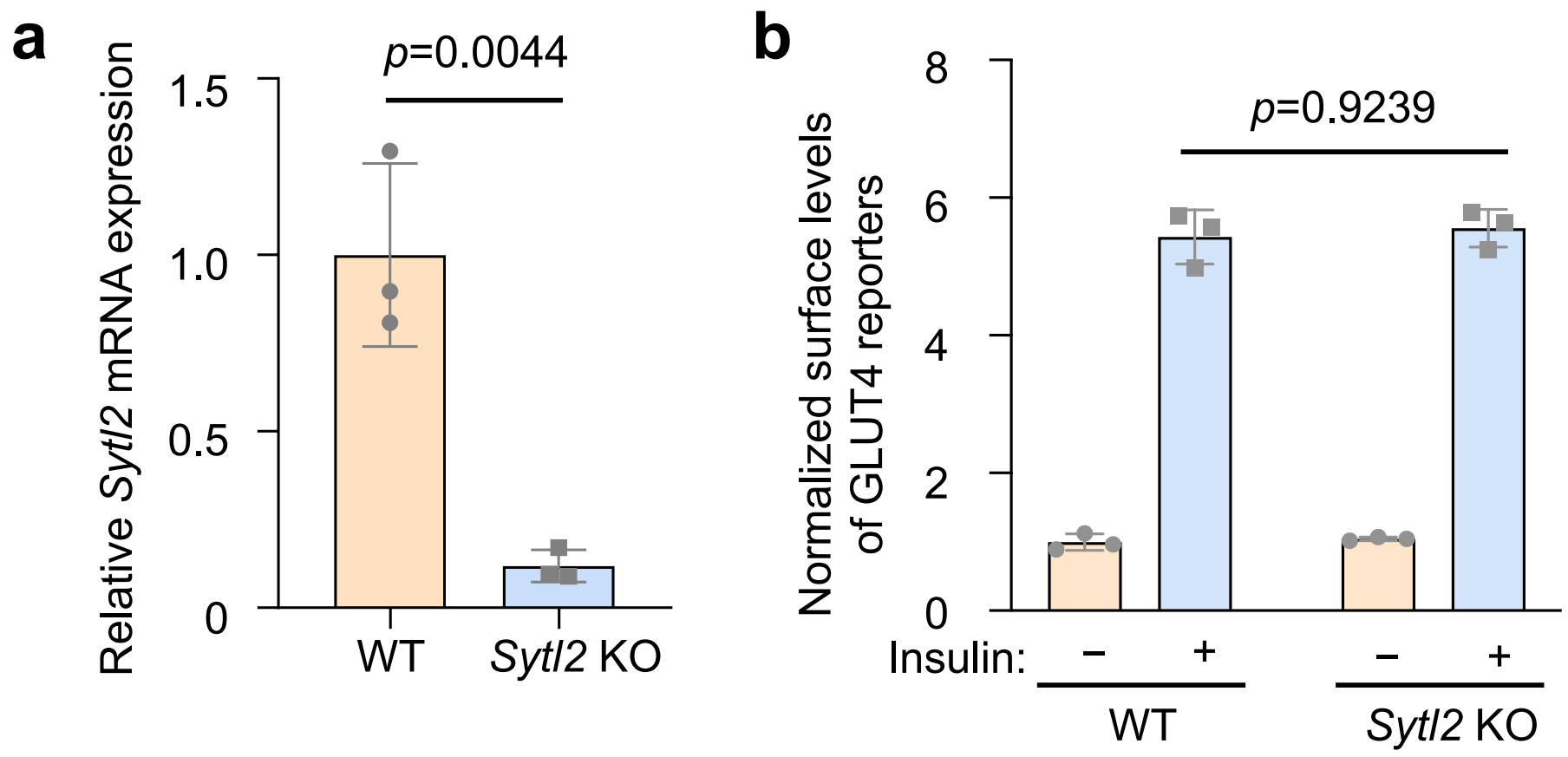

Supplementary Figure 7. Sytl2 is dispensable for insulin-stimulated GLUT4 exocytosis. (a) Relative expression levels of *Sytl2* mRNA in WT and *Sytl2* KO mouse preadipocytes measured by qRT-PCR using *Gapdh* as a reference mRNA. **(b)** Normalized surface levels of the HA-GLUT4-GFP reporter in the indicated adipocytes. Cells were either untreated or treated with 100 nM insulin for 30 minutes before surface reporter levels were measured using flow cytometry. Data are presented as mean ± SD of three biological replicates. *P* values were calculated using one-way ANOVA and Tukey's multiple comparison test. Related to Figure 2.

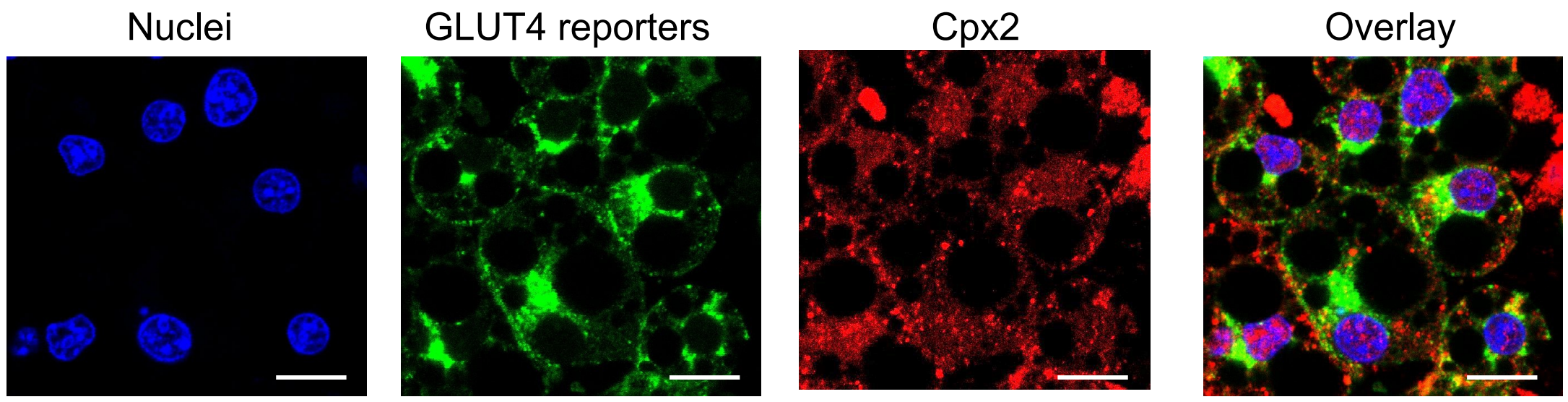

Supplementary Figure 8. Subcellular localization of Cpx2 in unstimulated adipocytes. WT mouse adipocytes expressing GLUT4 reporters (ALFA-GLUT4-mGreenLantern; PMID: 39145939) were fixed, permeabilized, and stained with anti-Cpx2 antibodies followed by Alexa Fluor 568-conjugated secondary antibodies. Nuclei were stained with Hoechst 33342. Cells were visualized under a confocal microscope. Representative confocal images from three independent experiments are shown. Scale bars, 10 µm. Related to Figure 3.

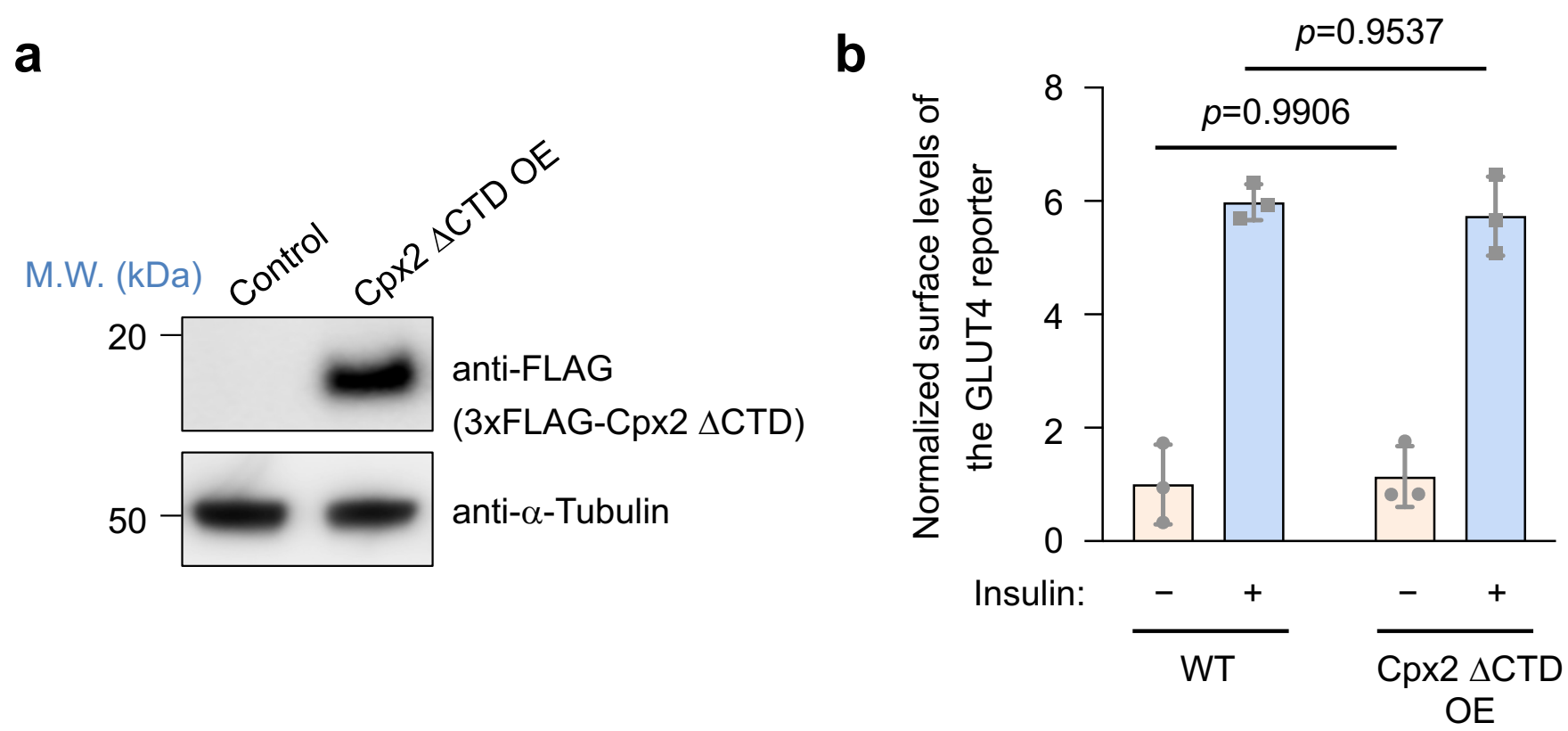

Supplementary Figure 9. Overexpression (OE) of a Cpx2 ΔCTD mutant does not alter basal surface levels of GLUT4 reporters. (a) Representative immunoblots from three independent experiments showing the expression of the indicated proteins. **(b)** Normalized surface levels of the HA-GLUT4-GFP reporter in the indicated mouse adipocytes. Cells were either untreated or treated with 100 nM insulin for 30 minutes before surface levels of the GLUT4 reporter were measured using flow cytometry. Data are presented as mean ± SD of three biological replicates. *P* values were calculated using two-way ANOVA and Tukey's multiple comparison test. Related to Figure 3.

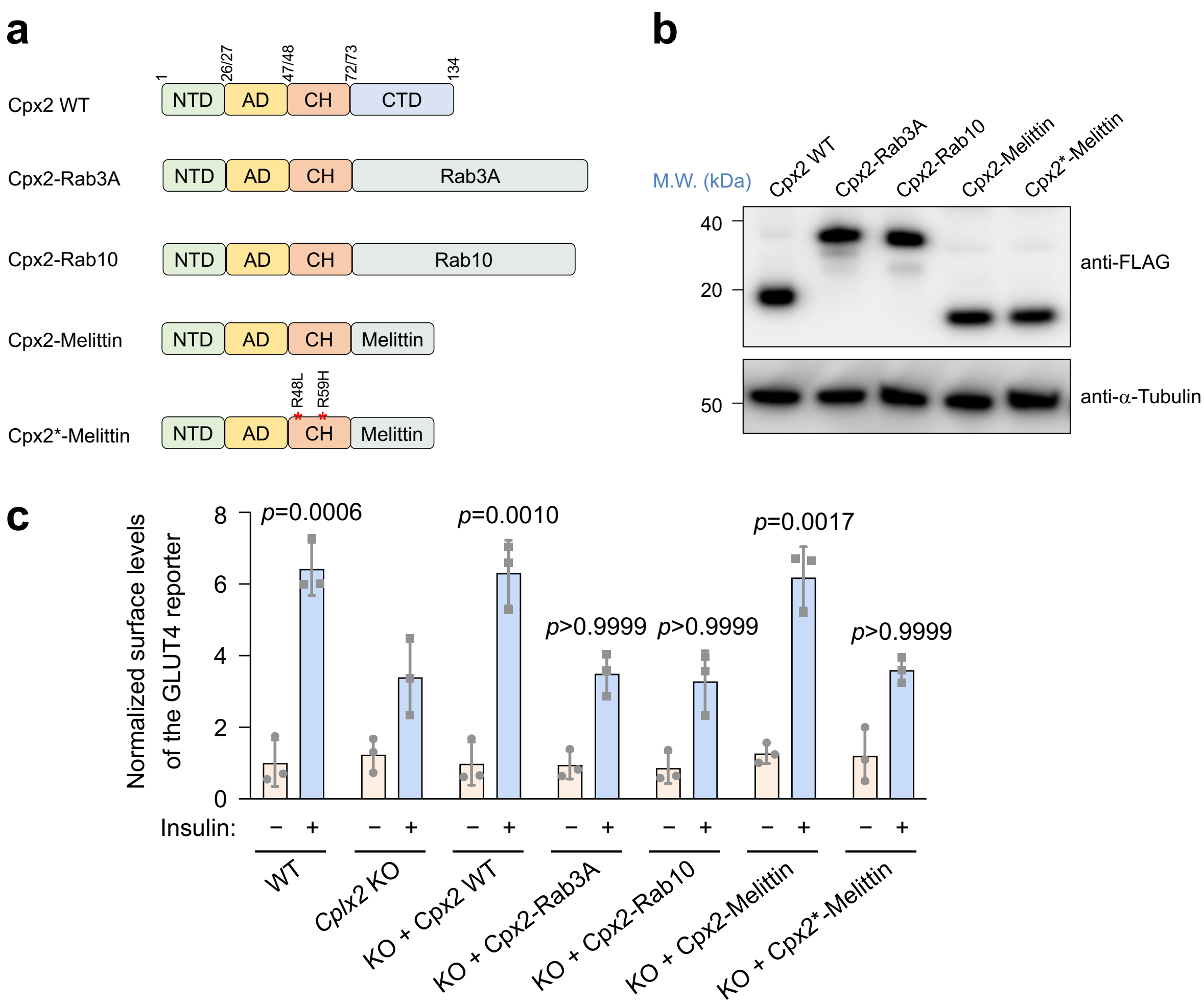

Supplementary Figure 10. Role of the C-terminal domain (CTD) of Cpx2 in Syt-independent exocytosis. (a) Diagrams of WT Cpx2 and Cpx2 chimeras in which the CTD was replaced with sequences from full-length (FL) mouse Rab3A, FL mouse Rab10, or melittin. All proteins were N-terminally tagged with a 3xFLAG epitope. The asterisk indicates a Cpx2 mutant bearing R48L and R59H substitutions. **(b)** Representative immunoblots from three independent experiments showing the expression of the indicated proteins in adipocytes. **(c)** Normalized surface levels of the HA-GLUT4-GFP reporter in WT adipocytes, *Cplx2* KO adipocytes, and KO adipocytes expressing the indicated proteins. Cells were either untreated or treated with 100 nM insulin for 30 minutes before surface GLUT4 reporter levels were measured using flow cytometry. Data are presented as mean ± SD of three biological replicates. Data from insulin-treated samples were compared to those from insulin-treated *Cplx2* KO adipocytes. *P* values were calculated using two-way ANOVA with Tukey's multiple comparison test. Related to Figure 6.
