# Supplementary material for: A primordial synaptotagmin-independent function of complexin in regulated exocytosis
Source: Nat Commun. 2026 Jun 27;17:8035. doi: 10.1038/s41467-026-74947-4 (PMC13454271; doi:10.1038/s41467-026-74947-4)
Supplement: Supplementary file 2 [file 41467_2026_74947_MOESM2_ESM.pdf]

Description of Additional Supplementary Files

Supplementary Data 1 - Proteomic analysis of mouse adipocytes.
Related to **Figure 1**.

Supplementary Data 2 - Proteomic analysis of the CAD neuronal progenitor cell line.
Related to **Figure 1**.
