# Supplementary material for: A primordial synaptotagmin-independent function of complexin in regulated exocytosis
Source: Nat Commun. 2026 Jun 27;17:8035. doi: 10.1038/s41467-026-74947-4 (PMC13454271; doi:10.1038/s41467-026-74947-4)
Supplement: Supplementary file 3 [file 41467_2026_74947_MOESM5_ESM.pdf]

Corresponding author(s): Jingshi Shen

Last updated by author(s): Apr 23, 2026

Reporting Summary

Nature Portfolio wishes to improve the reproducibility of the work that we publish. This form provides structure for consistency and transparency in reporting. For further information on Nature Portfolio policies, see our Editorial Policies and the Editorial Policy Checklist.

Please do not complete any field with "not applicable" or n/a. Refer to the help text for what text to use if an item is not relevant to your study.
For final submission: please carefully check your responses for accuracy; you will not be able to make changes later.

Statistics

For all statistical analyses, confirm that the following items are present in the figure legend, table legend, main text, or Methods section.

n/a	Confirmed	
☐	☒	The exact sample size (*n*) for each experimental group/condition, given as a discrete number and unit of measurement
☐	☒	A statement on whether measurements were taken from distinct samples or whether the same sample was measured repeatedly
☐	☒	The statistical test(s) used AND whether they are one- or two-sided<br>*Only common tests should be described solely by name; describe more complex techniques in the Methods section.*
☒	☐	A description of all covariates tested
☒	☐	A description of any assumptions or corrections, such as tests of normality and adjustment for multiple comparisons
☐	☒	A full description of the statistical parameters including central tendency (e.g. means) or other basic estimates (e.g. regression coefficient) AND variation (e.g. standard deviation) or associated estimates of uncertainty (e.g. confidence intervals)
☐	☒	For null hypothesis testing, the test statistic (e.g. *F*, *t*, *r*) with confidence intervals, effect sizes, degrees of freedom and *P* value noted<br>*Give P values as exact values whenever suitable.*
☒	☐	For Bayesian analysis, information on the choice of priors and Markov chain Monte Carlo settings
☒	☐	For hierarchical and complex designs, identification of the appropriate level for tests and full reporting of outcomes
☒	☐	Estimates of effect sizes (e.g. Cohen's *d*, Pearson's *r*), indicating how they were calculated

Our web collection on statistics for biologists contains articles on many of the points above.

Software and code

Policy information about availability of computer code

Data collection	The Western blots data: Bio-Rad ChemiDoc Imager<br>The Flow data: Beckman Coulter CyAn-ADP analyzer<br>Structure data: PDB 1KIL<br>Applied Biosystems™ 7500 Fast Real-Time PCR System: RT-qPCR<br>Images data: Nikon A100
Data analysis	UCSF ChimeraX-1.7, ImageJ-2.14, Flowjo-10.8, and Graphpad Prism-9.0

For manuscripts utilizing custom algorithms or software that are central to the research but not yet described in published literature, software must be made available to editors and reviewers. We strongly encourage code deposition in a community repository (e.g. GitHub). See the Nature Portfolio guidelines for submitting code & software for further information.

Data

Policy information about availability of data

All manuscripts must include a data availability statement. This statement should provide the following information, where applicable:

- Accession codes, unique identifiers, or web links for publicly available datasets
- A description of any restrictions on data availability
- For clinical datasets or third party data, please ensure that the statement adheres to our policy

Source data are provided in this paper. The in-silico screening data are available in Table 1. AlphaFold3-predicted structural models are included as Supplementary Data. Additional data are available from the corresponding authors upon reasonable request.

Research involving human participants, their data, or biological material

Policy information about studies with human participants or human data. See also policy information about sex, gender (identity/presentation), and sexual orientation and race, ethnicity and racism.

Reporting on sex and gender	not applicable
Reporting on race, ethnicity, or other socially relevant groupings	not applicable
Population characteristics	not applicable
Recruitment	not applicable
Ethics oversight	not applicable

Note that full information on the approval of the study protocol must also be provided in the manuscript.

Field-specific reporting

Please select the one below that is the best fit for your research. If you are not sure, read the appropriate sections before making your selection.

- [x] Life sciences
- [ ] Behavioural & social sciences
- [ ] Ecological, evolutionary & environmental sciences

For a reference copy of the document with all sections, see nature.com/documents/nr-reporting-summary-flat.pdf

Life sciences study design

All studies must disclose on these points even when the disclosure is negative.

Sample size	The sample sizes are clearly detailed in the figure legends.
Data exclusions	No data were excluded from the analyses.
Replication	All biological experiments were performed at least three times to ensure reproducibility. Western blot data were validated using a minimum of three independent samples.
Randomization	The randomization is clearly detailed in the figure legends.
Blinding	No blinding was used.

Reporting for specific materials, systems and methods

We require information from authors about some types of materials, experimental systems and methods used in many studies. Here, indicate whether each material, system or method listed is relevant to your study. If you are not sure if a list item applies to your research, read the appropriate section before selecting a response.

Materials & experimental systems

n/a	Involved in the study
	[x] Antibodies
	[x] Eukaryotic cell lines
[x]	Palaeontology and archaeology
[x]	Animals and other organisms
[x]	Clinical data
[x]	Dual use research of concern
[x]	Plants

Methods

n/a	Involved in the study
[x]	ChIP-seq
	[x] Flow cytometry
[x]	MRI-based neuroimaging

Antibodies

Antibodies used

Primary antibodies used in immunoblotting included anti-Cpx1/2 antibodies (Cell Signaling Technology, #28070, RRID: AB_2798954), anti-phospho-Akt (Ser473) antibodies (Cell Signaling Technology, #9271, RRID: AB_2716452), anti-Akt antibodies (Cell Signaling Technology, #9272, RRID: AB_329827), anti-peroxisome proliferator-activated receptor γ (Santa Cruz Biotechnology, #sc-7273, RRID: AB_628115), anti-AS160 antibodies (Cell Signaling Technology, #2670, RRID: AB_2199375), anti-phospho-AS160 (Thr642) antibodies (Cell Signaling Technology, #8881, RRID: AB_2651042), anti-Syt1 (clone m48), anti-Syt7 (Santa Cruz Biotechnology, #sc-293343) and anti-α-tubulin (DSHB, clone #12G10, RRID: AB_1210456). Secondary antibodies used in immunoblotting included horseradish peroxidase (HRP)-conjugated anti-rabbit antibodies (Sigma-Aldrich, #A6154, RRID: AB_258284), and HRP-conjugated anti-mouse antibodies (Sigma-Aldrich, #A6782, RRID: AB_258315). FLAG-tagged proteins were directly detected using HRP-conjugated anti-FLAG M2 antibodies (Sigma-Aldrich, #A8592, RRID: AB_439702). Secondary antibodies used in flow cytometry and imaging included Alexa Fluor 488-conjugated secondary antibodies (Thermo Fisher Scientific, #A11008, RRID: AB_142165), anti-mouse Alexa Fluor 568-conjugated secondary antibodies (Thermo Fisher Scientific, #A11004, RRID: AB_2534072), anti-rabbit Alexa Fluor 568-conjugated secondary antibodies (Thermo Fisher Scientific, #A11011, RRID: AB_143157).

Validation

These are commercial antibodies that have been widely used and reported in many publications.

Eukaryotic cell lines

Policy information about cell lines and Sex and Gender in Research

Cell line source(s)

HEK293T, preadipocytes

Authentication

HEK293T were purchased from ATCC. Preadipocytes are derived from inguinal white adipocyte tissues, a gift from Dr. Shingo Kajimura, Harvard University

Mycoplasma contamination

Cells were routinely tested for mycoplasma contamination with Hoechst 33342 and routinely maintained in Normocin (Invivogen)

Commonly misidentified lines
(See ICLAC register)

Not applicable

Plants

Seed stocks

Not applicable

Novel plant genotypes

Not applicable

Authentication

Not applicable

Flow Cytometry

Plots

Confirm that:

- [ ] The axis labels state the marker and fluorochrome used (e.g. CD4-FITC).
- [ ] The axis scales are clearly visible. Include numbers along axes only for bottom left plot of group (a 'group' is an analysis of identical markers).
- [ ] All plots are contour plots with outliers or pseudocolor plots.
- [x] A numerical value for number of cells or percentage (with statistics) is provided.

Methodology

Sample preparation	HEK 293T cells were cultured in Dulbecco's Modified Eagle Medium (DMEM) supplemented with 10% Fetal Bovine Serum (FBS, Sigma, #F0926) and penicillin/streptomycin (Corning, #130-002-CI). Immortalized mouse preadipocytes (derived from inguinal white adipocyte tissues, a gift from Dr. Shingo Kajimura, Harvard University) and HEK 293T cells were cultured in Dulbecco's Modified Eagle Medium (DMEM) supplemented with 10% Fetal Bovine Essence (FBE, VWR, #10803-034) and penicillin/streptomycin (Thermo Fisher Scientific, #15140122). All cell lines were maintained in a humidified 37 ºC incubator with 5% CO2. Mouse preadipocytes were differentiated into mature adipocytes using an established procedure. Preadipocytes were grown to ~95% confluence before a differentiation cocktail was added at the following final concentrations: 5 µg/mL insulin (Sigma-Aldrich, #I0516), 1 nM Triiodo-L-thyronine (T3, Sigma-Aldrich, #T2877), 125 µM indomethacin (Sigma-Aldrich, #I7378), 5 µM dexamethasone (Sigma-Aldrich, #D1756), and 0.5 mM 3-isobutyl-1-methylxanthine (IBMX, Sigma-Aldrich, #I5879). After two days, the cells were switched to DMEM supplemented with 10% FBE, 5 µg/mL insulin, and 1 nM T3. After another two days, cell culture media were replaced with fresh DMEM media supplemented with 10% FBE and 1 nM T3. Adipocytes were analyzed six days after switching to the differentiation cocktail. For flow cytometry, adipocytes cells were washed with KRH buffer (12 mM HEPES, pH 7.0, 121 mM NaCl, 4.9 mM KCl, 1.2 mM MgSO4, and 0.33 mM CaCl2) and and starved in KRH buffer for two hours. Cells were then incubated in KRH buffer containing insulin for the indicated durations, chilled on ice, and blocked with 5% FBE in KRH buffer at 4°C. Surface GLUT4 reporters were stained using anti-HA antibodies followed by APC-conjugated secondary antibodies. Cells were dissociated using Accutase and analyzed on a CyAN ADP analyzer (Beckman Coulter) or a MACSQuant Analyzer (Miltenyi Biotec). For lipid droplet staining and analysis.Preadipocytes were grown in clear-bottom 96-well plates. After differentiation, adipocytes were fixed with 4% PFA and stained with Oil Red O (Sigma-Aldrich, #O0625). Images of stained lipid droplets were captured using a bright-field microscope. In parallel, stained cells were air-dried at the room temperature for 2 hours, after which methanol was added to solubilize Oil Red O. Absorbance of the supernatant was measured at 520 nm on a plate reader.<br>For immunostaining and imaging, to visualize Cpx2, cells grown on coverslips were fixed using 4% PFA and permeabilized in PBS containing 5% FBE and 0.2% saponin. Endogenous Cpx2 was labeled using anti-Cpx1/2 primary antibodies (Cell Signaling Technology, #28070, RRID: AB_2798954) and Alexa Fluor 568-conjugated secondary antibodies (Thermo Fisher Scientific, #A11004, RRID: AB_2534072). To visualize surface GLUT4 reporters, preadipocytes cultured in four-chamber glass bottom dishes were differentiated into mature adipocytes, fixed with 4% paraformaldehyde, and blocked with 2% bovine serum albumin (BSA) without permeabilization. Surface reporters were stained with anti-HA antibodies and Alexa Fluor 568-conjugated secondary antibodies (Thermo Fisher Scientific, #A11004, RRID: AB_2534072). Nuclei were stained with Hoechst 33342 (Sigma-Aldrich, #D9642). For whole-cell proteomics, adipocytes and CAD cells were harvested in lysis buffer [100 mM triethyl ammonium bicarbonate (Millipore-Sigma, #18597), 5 mM tris (2-carboxyethyl) phosphine, 20 mM chloroacetamide (Millipore-Sigma, #C0267), and 1% SDS]. The samples were labeled using the TMTsixplex Isobaric Label Reagent Set (Thermo Fisher Scientific, #90061). Samples were prepared for mass spectrometry using the protein aggregation capture (PAC) method. For immunoblotting cells grown in 24-well plates were lysed in a SDS protein sample buffer (80 mM Tris, pH 6.8, 2% SDS, 10% glycerol, 0.0006% Bromophenol blue, and 0.1 M DTT). The samples were resolved on 8% Bis-Tris SDS-PAGE, transferred to PVDF membranes, and probed using indicated antibodies. For Quantitative reverse transcription PCR (qRT-PCR), total RNAs were isolated using a RNeasy Mini Kit (Qiagen, #74104). After treatment with ezDNAse (Thermo Fisher Scientific, #11766051), first strand complementary DNA was synthesized using a SuperScript IV kit (Thermo Fisher Scientific, #18091050). Gene expression was quantified using qRT-PCR on an Applied Biosystems™ 7500 Fast Real-Time PCR System using SsoAdvanced Universal SYBR Green Supermix (Bio-Rad, #172-5272) with gene-specific primer sets. Cycle threshold values of Sytl2 were normalized to those of Gapdh, which was used as a reference gene. Recombinant proteins were expressed in BL21 (DE3) E. coli (Stratagene, #230132) and purified using established procedures.
Instrument	Beckman Coulter CyAn ADP analyzer, Nikon A100
Software	ImageJ-2.14, Flowjo-10.8, and Graphpad Prism-9.0
Cell population abundance	Samples were not sorted; whole cells were analyzed by flow cytometry after dissociation.
Gating strategy	Cells were gated based on a clear population using FSC/SSC setting of CyAn, and ~5,000 cells were collected for analysis.

- [ ] Tick this box to confirm that a figure exemplifying the gating strategy is provided in the Supplementary Information.

This checklist template is licensed under a Creative Commons Attribution 4.0 International License, which permits use, sharing, adaptation, distribution and reproduction in any medium or format, as long as you give appropriate credit to the original author(s) and the source, provide a link to the Creative Commons license, and indicate if changes were made. The images or other third party material in this article are included in the article's Creative Commons license, unless indicated otherwise in a credit line to the material. If material is not included in the article's Creative Commons license and your intended use is not permitted by statutory regulation or exceeds the permitted use, you will need to obtain permission directly from the copyright holder. To view a copy of this license, visit http://creativecommons.org/licenses/by/4.0/
