# Supplementary material for: A primordial synaptotagmin-independent function of complexin in regulated exocytosis
Source: Nat Commun. 2026 Jun 27;17:8035. doi: 10.1038/s41467-026-74947-4 (PMC13454271; doi:10.1038/s41467-026-74947-4)
Supplement: Supplementary file 4 [file 41467_2026_74947_MOESM6_ESM.pdf]

nature portfolio

Peer Review File

A primordial synaptotagmin-independent function of complexin in regulated exocytosis

Corresponding Author: Professor Jingshi Shen

This file contains all reviewer reports in order by version, followed by all author rebuttals in order by version.

Version 0:

Reviewer comments:

Reviewer #1

(Remarks to the Author)
This manuscript identifies a physiologically relevant, synaptotagmin-independent function of Complexin in regulated exocytosis by demonstrating that Complexin is required for insulin stimulated GLUT4 vesicle fusion in adipocytes, a system reported to lack detectable synaptotagmin expression. Through a combination of genetic, cell biological, and biochemical approaches, the authors provide compelling evidence that Complexin functions as a positive regulator of vesicle fusion in this pathway, without an apparent clamping role, and that this activity requires both SNARE binding via the central helix and membrane engagement via the amphipathic C terminal domain. The systematic deletion, domain swap, and rescue experiments are particularly elegant and provide strong mechanistic insight. Overall, this work makes a significant conceptual contribution by defining a synaptotagmin independent role for complexin that may reflect an ancestral function of the protein and helps reconcile longstanding debates regarding the facilitatory versus inhibitory activities of complexin.

I am very positive about the significance and overall quality of the study. However, several issues related to mechanistic interpretation and the strength of certain claims should be addressed to further solidify the conclusions and clarify the broader implications of the findings.

1. The central premise relies on adipocytes lacking all synaptotagmin isoforms, thereby establishing a truly synaptotagmin independent exocytic pathway. The manuscript states that no synaptotagmin isoforms were detected by mass spectrometry and argues that sub detection levels are unlikely to be biologically meaningful. However, this conclusion is not supported by orthogonal validation. This point is particularly important because regulated exocytosis can be sensitive to low stoichiometric levels of key regulators. Targeted qRT-PCR and or immunoblotting for representative synaptotagmin isoforms would substantially strengthen the central premise.

2. The manuscript interprets Complexin as acting exclusively at the terminal membrane fusion step rather than earlier stages such as vesicle priming or maturation. In synaptic systems, Complexin has been implicated in both priming and fusion control, making this distinction conceptually important for assessing how generalizable the conclusions are. The evidence for a terminal fusion role is based largely on normal vesicle recruitment to the plasma membrane observed by TIRF and confocal microscopy. However, these approaches do not clearly distinguish defects in final priming from defects in membrane merger. In addition, GLUT4 surface measurements are performed at a single endpoint, 30 minutes after insulin application, and therefore do not provide information on fusion kinetics or fusion pore opening. The authors should either temper this conclusion or provide additional kinetic or functional assays that directly separate late priming from fusion pore formation. The language throughout the manuscript should be softened to state that Complexin acts at a late stage of exocytosis rather than unambiguously at the membrane merger step. Relatedly, statements referring to Complexin accelerating fusion, particularly in the abstract, should be revised or removed unless directly supported by kinetic measurements.

3. The effects of Complexin manipulation on basal GLUT4 surface levels are asymmetric and conceptually difficult to reconcile with the proposed model. Loss of Complexin does not reduce basal surface GLUT4, whereas over-expression increases basal surface GLUT4. If Complexin functions as a general activator of SNARE mediated fusion in this pathway, changes in its abundance would be expected to alter basal GLUT4 exocytosis in both directions, albeit with a larger dynamic range under insulin stimulated conditions. The observed asymmetry suggests that Complexin is not simply rate limiting for basal fusion, and instead may act downstream of, or in concert with, insulin dependent priming steps that

determine the availability of fusion competent vesicles. This conceptual issue is not currently addressed and weakens the claim that Complexin functions as a general fusion accelerator. This point could be strengthened by providing a mechanistic explanation for the asymmetric phenotypes, tempering the interpretation to explicitly frame Complexin as a conditionally engaged facilitator whose activity is gated by insulin signaling, or adding experiments that directly assess fusion kinetics or vesicle pool utilization under basal and stimulated conditions.

4. The manuscript demonstrates that the C terminal domain (CTD) of Complexin is essential for insulin stimulated GLUT4 exocytosis and argues that this domain promotes vesicle fusion by remodeling membranes. However, the CTD is known to serve multiple mechanistically distinct roles that are not fully disentangled in the current experiments. These include (i) preferential binding to highly curved vesicle membranes, which increases the effective local concentration of Complexin at fusion sites (Snead, D. et al. Nat Commun 2014, 5, 4955; Seiler et al. FEBS Lett 2009, 583, 2343-48; Gong, J. et al. Proc Natl Acad Sci U S A, 2016 113, E7590-99), (ii) spatial positioning of Complexin to gain privileged access to assembling SNARE complexes (Lottermoser et al. J Physiol 2025, 603.20, 6229-47), and (iii) direct perturbation of membrane structure that can promote lipid rearrangements during fusion pore formation and expansion (Courtney et al. Nat Struct Mol Biol. 2022, 29, 97-107). Because these functions are conceptually distinct, attributing the requirement for the CTD primarily to membrane remodeling risks oversimplifying the mechanism.

To disentangle these contributions, the authors could consider additional controls or clarifications. Testing whether over-expression of a CTD deletion mutant can partially rescue GLUT4 exocytosis would help determine whether increased Complexin abundance can compensate for loss of vesicle targeting. Evaluating vesicle specific membrane anchors, such as Rab3 based targeting (Lottermoser et al. J Physiol 2025, 603.20, 6229-47), rather than only generic membrane anchors, would test whether privileged positioning at vesicle fusion sites can substitute for the native C terminal domain.

There are also concerns regarding the melittin rescue strategy. Melittin is a highly potent amphipathic peptide known to induce membrane perturbation and pore formation. Although the rescue by the melittin fusion is striking, it could reflect nonspecific membrane destabilization rather than a mechanistically faithful replacement of the C terminal domain. Introducing SNARE binding disrupting mutations, such as R48L and R59H, into the melittin fused Complexin construct would test whether the rescue remains dependent on canonical SNARE engagement. Demonstrating SNARE dependence under these conditions would strengthen the conclusion that membrane remodeling acts in concert with SNARE binding rather than bypassing the normal fusion machinery.

Overall, the Discussion should also more explicitly acknowledge that the C-terminal domain likely integrates multiple functions and depending on the experimental outcomes, discuss which aspects are important for the facilitator function of Complexin in the cellular context examined.

5. Representative flow-cytometry gating and sorting data should be included in the Supplementary Figures to allow assessment of data quality and reproducibility.

Reviewer #2

(Remarks to the Author)
I co-reviewed this manuscript with one of the reviewers who provided the listed reports. This is part of the Nature Communications initiative to facilitate training in peer review and to provide appropriate recognition for Early Career Researchers who co-review manuscripts.

Reviewer #3

(Remarks to the Author)
The manuscript by Chen et al. defines a syntaptotagmin-independent function of complexin, Cpx2, in the insulin-regulated exocytosis of GLUT4-containing vesicles in adipocytes. The data show that, in this instance, Cpx acts as a positive regulator of exocytosis, which is distinct from its function as a negative regulator or clamp in the setting of synaptic vesicle exocytosis. The data are, in general, convincing, and the work will be of broad interest to investigators studying membrane trafficking and insulin action.

There are a few areas that could be more fully elucidated:

Is Cpx2 recruited to plasma membranes upon insulin stimulation? In unstimulated cells, does it reside in the cytosol, or on GLUT4 vesicles or at the plasma membrane? As a related question, is the Cpx2 CTD sufficient to bind membranes in liposome flotation assays? Is there a particular phospholipid species for which it has greatest affinity?

Data in Fig. 2e and 2f are from TIRF microscopy, but only the TIRF images are shown (Fig. 2e) and quantified (Fig. 2f), and only insulin-stimulated cells are shown. It would be helpful to show unstimulated and insulin-stimulated cells, to include the corresponding epifluorescence images, and to quantify the ratio of TIRF:epifluorescence image intensities. This would help to support the idea that the vesicles are docked but not fused at the cell surface.

Several other points need to be clarified or strengthened:

In the legend for Fig. 1, and in subsequent figures, the work relies on “mouse adipocytes,” but these cells are apparently not primary adipocytes isolated from mice. This is confusing. The methods section refers to “immortalized mouse preadipocytes,” but the source of these cells is unclear. How were the cells immortalized? One of the references cited for the differentiation protocol states that cells were a gift from Shingo Kajimura, but no reference is cited. These details need to be clarified. Is there a better term that can be used to refer to the cells? At least the term “3T3-L1 adipocytes” indicates that cells were differentiated from an established cell line, and that they are not primary adipocytes. The term “mouse adipocytes” implies that they are primary adipocytes from mice, which is not the case here.

The investigators made Cplx1/2 double KO cells, in addition to the Cplx2 KO cells, and show that Cplx1 does not contribute further to the phenotype in Fig. 2c. If Cplx1 protein was undetectable in control cells, then how was the Cplx1 KO validated? Can qPCR be done for both Cplx2 and Cplx1? Is Cplx1 expressed in the undifferentiated cells and is it deleted in that case?

Quantification of GLUT4 exocytosis was done using flow cytometry, and the arithmetic mean of APC and GFP fluorescence intensities were used for calculations. Yet, such data often have a log-normal distribution, in part because lentiviral or retroviral expression of the reporter is best approximated by a logarithmic distribution, and data are typically acquired on a logarithmic scale using the flow cytometer. Thus, the median or geometric mean may be the more appropriate measure of central tendency (see PMIDs 11416153, 17967900). Presumably, background fluorescence intensities were subtracted, using GFP-negative cells and cells that were not stained using APC, respectively; these details should be described in the methods section.

In Fig. 4, GST-V2CD is not defined. Presumably this is a GST fusion protein with VAMP2, but what does “CD” indicate?

The image in Fig. 5c is rather light. Clearly, the Cpx2 deltaCTD* protein containing the point mutations binds less, compared to that without the point mutations, but it is not certain if it is absent or just reduced.

Version 1:

Reviewer comments:

Reviewer #1

(Remarks to the Author)
The authors have addressed all my concerns and make adequate revisions in the manuscript. I support the publication of the manuscript in the current form.

Reviewer #2

(Remarks to the Author)
I co-reviewed this manuscript with one of the reviewers who provided the listed reports. This is part of the Nature Communications initiative to facilitate training in peer review and to provide appropriate recognition for Early Career Researchers who co-review manuscripts.

Reviewer #3

(Remarks to the Author)
The authors have addressed my comments comprehensively. I support publication of this revised manuscript.

Open Access This Peer Review File is licensed under a Creative Commons Attribution 4.0 International License, which permits use, sharing, adaptation, distribution and reproduction in any medium or format, as long as you give appropriate credit to the original author(s) and the source, provide a link to the Creative Commons license, and indicate if changes were made.
In cases where reviewers are anonymous, credit should be given to 'Anonymous Referee' and the source.
The images or other third party material in this Peer Review File are included in the article's Creative Commons license, unless indicated otherwise in a credit line to the material. If material is not included in the article's Creative Commons license and your intended use is not permitted by statutory regulation or exceeds the permitted use, you will need to obtain permission directly from the copyright holder.
To view a copy of this license, visit https://creativecommons.org/licenses/by/4.0/

Point-by-point responses to Reviewers' comments

<u>Reviewer 1</u>

"1. The central premise relies on adipocytes lacking all synaptotagmin isoforms, thereby establishing a truly synaptotagmin independent exocytic pathway. The manuscript states that no synaptotagmin isoforms were detected by mass spectrometry and argues that sub detection levels are unlikely to be biologically meaningful. However, this conclusion is not supported by orthogonal validation. This point is particularly important because regulated exocytosis can be sensitive to low stoichiometric levels of key regulators. Targeted qRT-PCR and or immunoblotting for representative synaptotagmin isoforms would substantially strengthen the central premise."

<u>Response</u>: The Reviewer raised an excellent point. The absence of synaptotagmins (Syts) in adipocytes is supported by both our proteomic analysis and the lack of calcium-dependent regulation of exocytosis in adipocytes. However, we fully agree with the Reviewer that orthogonal validation is necessary. In response to the Reviewer's suggestion, we performed immunoblot analysis to examine the expression of Syt isoforms implicated in exocytosis. While these Syts are readily detected in neuronal progenitor cells, they are not detectable in mouse adipocytes. These results are consistent with our proteomic data and further support the conclusion that adipocytes lack Syt expression. The new data are presented in Supplementary Figure 1 of the revised manuscript. We thank the Reviewer for this important suggestion.

"2. The manuscript interprets Complexin as acting exclusively at the terminal membrane fusion step rather than earlier stages such as vesicle priming or maturation. In synaptic systems, Complexin has been implicated in both priming and fusion control, making this distinction conceptually important for assessing how generalizable the conclusions are. The evidence for a terminal fusion role is based largely on normal vesicle recruitment to the plasma membrane observed by TIRF and confocal microscopy. However, these approaches do not clearly distinguish defects in final priming from defects in membrane merger. In addition, GLUT4 surface measurements are performed at a single endpoint, 30 minutes after insulin application, and therefore do not provide information on fusion kinetics or fusion pore opening. The authors should either temper this conclusion or provide additional kinetic or functional assays that directly separate late priming from fusion pore formation. The language throughout the manuscript should be softened to state that Complexin acts at a late stage of exocytosis rather than unambiguously at the membrane merger step. Relatedly, statements referring to Complexin accelerating fusion, particularly in the abstract, should be revised or removed unless directly supported by kinetic measurements."

<u>Response</u>: The Reviewer correctly pointed out that while our data support that Cpx functions at a late stage of the GLUT4 exocytosis pathway, they do not resolve its precise step within this process. Based on the Reviewer's suggestion, we revised the manuscript throughout – including the Abstract, Results, and Discussion – to soften our conclusions and state that complexin acts at a late stage of exocytosis, rather than specifically at the membrane merger step. These revisions more accurately represent the scope of our data. We thank the Reviewer for improving the accuracy and clarity of the manuscript in this regard.

"3. The effects of Complexin manipulation on basal GLUT4 surface levels are asymmetric and conceptually difficult to reconcile with the proposed model. Loss of Complexin does not reduce basal surface GLUT4, whereas over-expression increases basal surface GLUT4. If Complexin functions as a general activator of SNARE mediated fusion in this pathway, changes in its abundance would be expected to alter basal GLUT4 exocytosis in both directions, albeit with a larger dynamic range under insulin stimulated conditions. The observed asymmetry suggests that Complexin is not simply rate limiting for basal fusion, and instead may act downstream of, or in concert with, insulin dependent priming steps that determine the availability of fusion competent vesicles. This conceptual issue is not currently addressed and weakens the claim that Complexin functions as a general fusion accelerator. This point could be strengthened by providing a mechanistic explanation for the asymmetric phenotypes, tempering the interpretation to explicitly frame Complexin as a conditionally engaged facilitator whose activity is gated by insulin signaling, or adding experiments that directly assess fusion kinetics or vesicle pool utilization under basal and stimulated conditions."

<u>Response</u>: The Reviewer correctly pointed out that the asymmetric effects on basal GLUT4 surface levels indicate that Cpx2 is not simply a rate-limiting activator of basal exocytosis. We agree that this asymmetry requires clarification. Our data suggest that Cpx2 acts on a pool of vesicles that becomes fusion-competent upon insulin stimulation. Under basal conditions, the availability of fusion-ready GLUT4 vesicles is limited by upstream insulin-dependent priming steps, such that loss of Cpx2 does not further reduce basal surface levels. In contrast, overexpression of Cpx2 may enhance the efficiency of fusion for vesicles that stochastically reach a fusion-competent state, thereby modestly increasing basal surface GLUT4. These findings indicate that Cpx2 is not a constitutive driver of basal exocytosis but instead functions at a late stage of the pathway that is engaged when vesicles become fusion-competent. Based on the Reviewer's suggestion, we revised the text to clarify that Cpx2 acts as a conditionally engaged facilitator of late-stage exocytosis (page 9, paragraph 2).

"4. The manuscript demonstrates that the C terminal domain (CTD) of Complexin is essential for insulin stimulated GLUT4 exocytosis and argues that this domain promotes vesicle fusion by remodeling membranes. However, the CTD is known to serve multiple mechanistically distinct roles that are not fully disentangled in the current experiments. These include (i) preferential binding to highly curved vesicle membranes, which increases the effective local concentration of Complexin at fusion sites (Snead, D. et al. Nat Commun 2014, 5, 4955; Seiler et al. FEBS Lett 2009, 583, 2343-48; Gong, J. et al. Proc Natl Acad Sci U S A, 2016 113, E7590-99), (ii) spatial positioning of Complexin to gain privileged access to assembling SNARE complexes (Lottermoser et al. J Physiol 2025, 603.20, 6229-47), and (iii) direct perturbation of membrane structure that can promote lipid rearrangements during fusion pore formation and expansion (Courtney et al. Nat Struct Mol Biol. 2022, 29, 97-107). Because these functions are conceptually distinct, attributing the requirement for the CTD primarily to membrane remodeling risks oversimplifying the mechanism.

To disentangle these contributions, the authors could consider additional controls or clarifications. Testing whether over-expression of a CTD deletion mutant can partially rescue GLUT4 exocytosis would help determine whether increased Complexin abundance can compensate for loss of vesicle targeting. Evaluating vesicle specific membrane anchors, such as Rab3 based targeting (Lottermoser et al. J Physiol 2025, 603.20, 6229-47), rather than only generic membrane

anchors, would test whether privileged positioning at vesicle fusion sites can substitute for the native C terminal domain.

There are also concerns regarding the melittin rescue strategy. Melittin is a highly potent amphipathic peptide known to induce membrane perturbation and pore formation. Although the rescue by the melittin fusion is striking, it could reflect nonspecific membrane destabilization rather than a mechanistically faithful replacement of the C terminal domain. Introducing SNARE binding disrupting mutations, such as R48L and R59H, into the melittin fused Complexin construct would test whether the rescue remains dependent on canonical SNARE engagement. Demonstrating SNARE dependence under these conditions would strengthen the conclusion that membrane remodeling acts in concert with SNARE binding rather than bypassing the normal fusion machinery.

Overall, the Discussion should also more explicitly acknowledge that the C-terminal domain likely integrates multiple functions and depending on the experimental outcomes, discuss which aspects are important for the facilitator function of Complexin in the cellular context examined."

Response: The Reviewer raised several crucial points regarding the CTD of complexin. To fully address these points, we performed the experiments suggested by the Reviewer.

First, we overexpressed a CTD deletion mutant of Cpx2 in WT adipocytes. In contrast to WT Cpx2 overexpression, overexpression of this mutant did not enhance basal surface levels of GLUT4 (Supplementary Fig. 9), indicating that the CTD is required for the exocytosis-stimulating activity of Cpx2 and that increased protein levels cannot compensate for loss of the CTD.

Second, to examine whether vesicle-specific localization can substitute for the CTD, we generated Cpx2-Rab3A and Cpx2-Rab10 chimeras, which target complexin to exocytic vesicles. However, neither chimera restored GLUT4 exocytosis when expressed at levels comparable to WT Cpx2 in *Cplx2* KO adipocytes (Supplementary Fig. 10), indicating that vesicle targeting alone is insufficient to support Cpx2 function. These results are consistent with those from other Cpx2 chimeras in which Cpx2 was anchored to membranes via the v-SNARE or KRAS, which also failed to rescue exocytosis (Fig. 6).

Third, we introduced SNARE-binding–disrupting mutations (R48L/R59H) into the Cpx2–melittin chimera. This mutant failed to support insulin-stimulated GLUT4 exocytosis (Supplementary Fig. 10), demonstrating that the melittin-mediated rescue remains dependent on canonical SNARE interactions rather than bypassing the fusion machinery.

Together, these experiments indicate that the CTD of Cpx2 plays a role in GLUT4 exocytosis beyond simple membrane anchoring. Based on the Reviewer's suggestion, we also revised the Discussion section to more explicitly acknowledge that the CTD likely integrates multiple functions and to clarify which aspects are supported by our data (page 15, paragraph 2).

"5. Representative flow-cytometry gating and sorting data should be included in the Supplementary Figures to allow assessment of data quality and reproducibility."

Response: Based on the Reviewer's suggestion, we included representative flow-cytometry gating and sorting data in Supplementary Figure 3 of the revised manuscript.

Finally, we'd like to thank the Reviewer again for sharing his/her deep expertise in complexin biology and regulated exocytosis; the manuscript has been significantly strengthened as a result of these revisions.

Reviewer 2

"I co-reviewed this manuscript with one of the reviewers who provided the listed reports. This is part of the Nature Communications initiative to facilitate training in peer review and to provide appropriate recognition for Early Career Researchers who co-review manuscripts."

Response: We have addressed the comments raised by the Reviewer in our responses to the co-reviewer's report and have revised the manuscript accordingly. We thank the Reviewer for the thoughtful and constructive feedback.

Reviewer 3

"Is Cpx2 recruited to plasma membranes upon insulin stimulation? In unstimulated cells, does it reside in the cytosol, or on GLUT4 vesicles or at the plasma membrane? As a related question, is the Cpx2 CTD sufficient to bind membranes in liposome flotation assays? Is there a particular phospholipid species for which it has greatest affinity?"

Response: The Reviewer raised several important questions regarding the localization and membrane binding of complexin. First, we further characterized the subcellular localization of Cpx2 under basal conditions. In unstimulated adipocytes, Cpx2 is not cytosolic but instead localizes to intracellular membrane compartments, where it partially colocalizes with GLUT4 reporters and shows minimal enrichment at the plasma membrane. These new data are included in Supplementary Figure 8. Upon insulin stimulation, Cpx2 becomes significantly enriched in the plasma membrane–proximal region, as shown by TIRF microscopy (Fig. 3e–f), indicating stimulus-dependent recruitment during the evoked phase of exocytosis.

Next, based on the Reviewer's suggestion, we revised the text to more clearly describe the biochemical properties of the CTD based on our liposome co-flotation assays, in the context of GLUT4 exocytosis, together with previous studies (page 11, paragraph 2). The amphipathic complexin CTD is both required and sufficient for binding to protein-free liposomes, with negatively charged lipids such as phosphatidylserine (PS) playing important roles in this interaction. These biochemical findings are consistent with our genetic analysis of the CTD in the GLUT4 exocytic pathway. We thank the Reviewer for these important suggestions.

"Data in Fig. 2e and 2f are from TIRF microscopy, but only the TIRF images are shown (Fig. 2e) and quantified (Fig. 2f), and only insulin-stimulated cells are shown. It would be helpful to show unstimulated and insulin-stimulated cells, to include the corresponding epifluorescence images, and to quantify the ratio of TIRF:epifluorescence image intensities. This would help to support the idea that the vesicles are docked but not fused at the cell surface."

Response: Based on the Reviewer's advice, we expanded our imaging analysis to further characterize GLUT4 translocation upon insulin stimulation. Specifically, we included both unstimulated and insulin-stimulated conditions, as well as corresponding epifluorescence images to assess total cellular GLUT4 reporter levels. We also quantified the relationship between TIRF and

epifluorescence signals. We find that in *Cplx2* KO cells, GLUT4 reporters accumulate normally in the TIRF field, while total GLUT4 levels (measured by epifluorescence) remain unchanged. These new data indicate that vesicles are efficiently recruited to the vicinity of the cell surface but are impaired at a step downstream of arrival, consistent with a role for Cpx2 at a late stage of the GLUT4 exocytic pathway (Fig. 2e–f). We thank the Reviewer for improving the clarity and rigor of the manuscript in this regard.

"In the legend for Fig. 1, and in subsequent figures, the work relies on "mouse adipocytes," but these cells are apparently not primary adipocytes isolated from mice. This is confusing. The methods section refers to "immortalized mouse preadipocytes," but the source of these cells is unclear. How were the cells immortalized? One of the references cited for the differentiation protocol states that cells were a gift from Shingo Kajimura, but no reference is cited. These details need to be clarified. Is there a better term that can be used to refer to the cells? At least the term "3T3-L1 adipocytes" indicates that cells were differentiated from an established cell line, and that they are not primary adipocytes. The term "mouse adipocytes" implies that they are primary adipocytes from mice, which is not the case here."

Response: Based on the Reviewer's advice, we revised the figure legends in both the main text and the Supporting Information, as well as the Methods section to explicitly state that these cells are adipocytes differentiated from an immortalized mouse preadipocyte cell line. We also clarified the origin and culture history of these cells in the Methods section.

"The investigators made Cplx1/2 double KO cells, in addition to the Cplx2 KO cells, and show that Cplx1 does not contribute further to the phenotype in Fig. 2c. If Cplx1 protein was undetectable in control cells, then how was the Cplx1 KO validated? Can qPCR be done for both Cplx2 and Cplx1? Is Cplx1 expressed in the undifferentiated cells and is it deleted in that case?"

Response: We thank the Reviewer for raising this intriguing point. As noted, Cpx1 protein is undetectable by immunoblotting in both preadipocytes and differentiated adipocytes, consistent with our proteomic data. Accordingly, conventional protein-level validation of the *Cplx1* gene KO is not feasible. In our CRISPR KO experiments, two gRNAs were used to simultaneously target two exon regions of a candidate gene, effectively disrupting gene function. To further address the Reviewer's point, we employed a well-established surrogate approach to validate CRISPR targeting efficiency for non-detectable proteins. Specifically, we tested whether the same gRNAs could efficiently target and eliminate expression of a *Cplx1* transgene. We observed that these gRNAs completely eliminated Cpx1 protein expression from the transgene, demonstrating their effectiveness in targeting the *Cplx1* locus. These data are now included in Supplementary Figure 2.

"Quantification of GLUT4 exocytosis was done using flow cytometry, and the arithmetic mean of APC and GFP fluorescence intensities were used for calculations. Yet, such data often have a log-normal distribution, in part because lentiviral or retroviral expression of the reporter is best approximated by a logarithmic distribution, and data are typically acquired on a logarithmic scale using the flow cytometer. Thus, the median or geometric mean may be the more appropriate measure of central tendency (see PMIDs 11416153, 17967900). Presumably, background fluorescence

intensities were subtracted, using GFP-negative cells and cells that were not stained using APC, respectively; these details should be described in the methods section."

Response: As correctly noted by the Reviewer, background fluorescence was subtracted from all measurements, and we revised the Methods section to clarify these procedures. We agree that flow cytometry data often follow a log-normal distribution and can be summarized using a range of statistical measures. In this study, we primarily used the arithmetic mean because it consistently reflected insulin-stimulated GLUT4 exocytosis across our previous genetic studies and showed strong correlation with independent imaging-based measurements.

To further address the Reviewer's suggestion, we re-analyzed representative datasets using both the median and geometric mean of fluorescence intensities. As shown in Supplementary Figure 4, these alternative analyses yielded results and statistical conclusions consistent with those obtained using the arithmetic mean. We thank the Reviewer for this important advice.

"In Fig. 4, GST-V2CD is not defined. Presumably this is a GST fusion protein with VAMP2, but what does "CD" indicate?"

Response: We apologize for the confusion. We revised the Figure 4 legend and Methods section to clarify that "CD" refers to the cytosolic domain.

"The image in Fig. 5c is rather light. Clearly, the Cpx2 deltaCTD protein containing the point mutations binds less, compared to that without the point mutations, but it is not certain if it is absent or just reduced."*

Response: In response to the Reviewer's comment, we added a new panel (Fig. 5d) showing the quantification of protein intensities on SDS-PAGE based on multiple independent experiments. These data demonstrate that the point mutations diminished the binding of Cpx2 to the SNARE complex in liposome co-flotation assays, consistent with our genetic results.

Finally, we would like to thank the Reviewer again for these excellent suggestions, which have substantially improved the quality of this manuscript.
